# S-ketamine mitigates paclitaxel-induced pain-related anxiety-like behavior through downregulation of mGluR5 and activation of the BDNF/TrkB signaling pathway

**DOI:** 10.3389/fneur.2026.1801549

**Published:** 2026-04-23

**Authors:** Jing Cao, ZiYuan Wang, GeGe Lv, XingLiao Luo, MengYue Wang, JingYu Hui, MengMeng Li, Yong Yang, YongHong Yang, Yi Hu, Qiang Lin

**Affiliations:** 1 Department of Anesthesiology, North China Petroleum Bureau General Hospital, Hebei Medical University, Renqiu, China; 2 Department of Anesthesiology, The First Affiliated Hospital of Zhengzhou University, Zhengzhou, China; 3 Graduate School, Hebei University of Chinese Medicine, Shijiazhuang, China; 4 Graduate School, Hebei North University, Zhangjiakou, China; 5 Department of Rehabilitation, North China Petroleum Bureau General Hospital, Hebei Medical University, Renqiu, China; 6 Department of Oncology, North China Petroleum Bureau General Hospital, Hebei Medical University, Renqiu, China

**Keywords:** anxiety, BDNF–TrkB, medial prefrontal cortex, mGluR5, paclitaxel, S-ketamine

## Abstract

**Background:**

Paclitaxel (PTX), a broad-spectrum anti-tumor drug, is extensively employed as a first-line chemotherapy for solid tumors, including lung and breast cancers. However, it induces chemotherapy-related pain and anxiety in over 53% of patients. S-ketamine, the S-enantiomer of ketamine, exerts rapid antidepressant effects. In this study, we established a PTX model to examine the effects of S-ketamine on pyramidal neurons in the prelimbic cortex (PrL) and the brain-derived neurotrophic factor (BDNF)/tropomyosin receptor kinase B (TrkB) pathway, specifically investigating the role of metabotropic glutamate receptor 5 (mGluR5).

**Methods:**

A PTX-induced peripheral neuropathy and pain-related anxiety mouse model was established by intraperitoneal injection of PTX. Mechanical allodynia was assessed using the electronic von Frey test. Anxiety-like behaviors were evaluated using the elevated plus maze (EPM) and open field test (OFT) after S-ketamine treatment. After behavioral testing, electrophysiological recordings, immunofluorescence staining, and Western blotting analyses were performed, specifically targeting the PrL subregion of the medial prefrontal cortex (mPFC).

**Results:**

Compared with PTX-treated mice, S-ketamine administration resulted in significant improvements across behavioral, molecular, and electrophysiological dimensions: significantly increased mechanical withdrawal thresholds indicated alleviated neuropathic pain; Increased central area distance/total distance ratio in the OFT and prolonged open arm time in the EPM demonstrated reduced anxiety-like behaviors; concomitant decreases in mGluR5 expression and pyramidal neuron firing rates were observed alongside enhanced theta-gamma phase-amplitude coupling, and upregulation of BDNF and TrkB expression in the PrL was detected.

**Conclusion:**

S-ketamine mitigates PTX-induced mechanical allodynia and anxiety-like behaviors, an effect that is closely associated with the downregulation of mGluR5 and the concurrent modulation of the BDNF/TrkB signaling pathway in the PrL of the mPFC.

## 1 Introduction

Paclitaxel (PTX) serves as a broad-spectrum antineoplastic agent and is extensively utilized in first-line chemotherapy for solid tumors, including breast and lung cancers (1, 2). However, PTX-associated chemotherapy-induced peripheral neuropathy (CIPN) is a severe neurogenic pain condition that affects 60–70% of patients, posing a major clinical challenge for neurologists. Its progressive hyperalgesia and sensory disturbances being its predominant clinical manifestations (3). Approximately 80% of PTX-treated patients develop persistent neuropathic pain, characterized by abnormal pain responses to non-noxious stimuli, with some experiencing lifelong pain hypersensitivity (4–6). The patients’ quality of life is severely affected by this chronic pain. Evidence from clinical cohorts indicates that over 53% of patients with CIPN develop persistent neuropsychiatric comorbidities, including fear-related distress and clinical anxiety (7). This pain-anxiety comorbidity exhibits bidirectional pathophysiology; neuropathic pain induces limbic dysregulation through central sensitization mechanisms, and anxiety disorders amplify pain perception through reduced nociceptive thresholds, forming a clinically significant vicious cycle (8). Accordingly, a comprehensive investigation of the brain mechanisms underlying PTX-induced neurogenic pain-related anxiety is crucial for neurologists to develop targeted interventions and enhance the quality of life of cancer patients.

The medial prefrontal cortex (mPFC), particularly its prelimbic (PrL) subregion, is an evolutionarily conserved region mediating pain and affective processing in rodents and humans, which undergoes structural and functional plasticity in neuropathic pain conditions (9). Metabotropic glutamate receptors (mGluRs) are synaptic glutamate-sensing G protein-coupled receptors (GPCRs), and alterations in their signaling activity are strongly implicated in the dysregulation of neuronal circuits associated with anxiety (10). Specifically, the pharmacological antagonism or negative allosteric modulation of metabotropic glutamate receptor 5 (mGluR5) has emerged as a promising therapeutic strategy for anxiety disorders, as evidenced by preclinical and clinical studies (11). By upregulating mGluR5 in the PrL, PTX enhances glutamatergic neurotransmission to two critical targets, the spinal dorsal horn (causing neuropathic pain and central sensitization) and the basolateral amygdala (potentiating anxiety-like behaviors), establishing a neural substrate for pain-related anxiety (12). Furthermore, evidence from other models of neuropathic pain and affective disorders indicates that aberrant mGluR5 overexpression can suppress brain-derived neurotrophic factor (BDNF) levels (13). As BDNF-tropomyosin receptor kinase B (TrkB) signaling is essential for synaptic resilience, its impairment disrupts cortical inhibition and perpetuates pain-anxiety crosstalk (14). Consequently, we hypothesized that mGluR5/NMDAR-driven mPFC hyperexcitability coupled with impaired BDNF–TrkB neurotrophic support might create a self-sustaining circuit pathology, exacerbating both sensory hyperalgesia and pain-related affective disorders in PTX-treated rodents.

S-ketamine, an enantiomer of (R, S)-ketamine (ketamine), demonstrates rapid antidepressant effects and reverses chronic stress-induced synaptic deficits in the PrL by promoting dendritic spine formation (15). S-ketamine antagonizes NMDA receptors and downregulates mGluR5, potentially reducing glutamatergic hyperactivity in the PrL-basolateral amygdala (BLA) circuit implicated in affective disorders (16, 17). It significantly modulates BDNF levels and glutamatergic synaptic properties in the PrL, enhancing synaptic plasticity to produce analgesic and anxiolytic effects (18). Surgical stress exacerbates pain hypersensitivity and anxiety in patients undergoing chemotherapy, thereby impairing their postoperative recovery (19). Given the efficacy of esketamine in anesthesia and treatment-resistant depression, adjunctive intraoperative infusion may mitigate postoperative pain and comorbid anxiety in patients receiving neoadjuvant chemotherapy (20). However, the extent of its anxiolytic effects and underlying mechanisms remains unclear, necessitating further investigation into esketamine’s actions against chemotherapy-induced pain-related anxiety to inform clinical translation.

## 2 Methods

### 2.1 Animals

The Animal Ethics Committee of North China Petroleum Bureau General Hospital approved all experimental procedures used in this study (Approval No: hbyyec-kysb-202305) and ensured that they were performed according to the Animal Research: Reporting of *In Vivo* Experiments (ARRIVE) guidelines and animal ethics principles. Adult male C57BL/6 mice (8–12 weeks old, 20–25 g) were acquired from Liaoning Changsheng Biotechnology Co., Ltd. The animal license number was SCXK (Liao) 2020–0001. The animals were housed in groups of 4–5 per cage under the following conditions: Free access to food and water, a 12-h light/dark cycle (lights on at 7:00 a.m.), a room temperature of 22–25 °C, air humidity of 50–60%, and daily feed variations. In both stages of the experiments, the sample size was 12 mice per group (*n* = 12) for all behavioral assessments. Notably, within these 12 mice, a randomly selected subset of 3 mice (*n* = 3 per group) had been pre-implanted with microelectrodes, and their *in vivo* local field potentials were synchronously recorded during the behavioral tests. Following the completion of all behavioral assessments, to minimize animal usage in accordance with the 3R principles, the 12 mice in each group were randomly allocated for downstream tissue analyses: 6 mice were used for Western blotting (*n* = 6), and the remaining 6 mice were used for immunofluorescence staining (*n* = 6).

### 2.2 Pain model and group assignment

The PTX model (21) was used to simulate the pain caused by chemotherapy. The experiment was conducted in two stages. 2-Chloro-5-hydroxybenzylglycine (CHPG) is an agonist for mGluR5 (22). In the first stage, mice were randomly divided into the following four groups: Vehicle, PTX, S-ketamine, and PTX + S-ketamine. In the second stage, the mice were randomly divided into two groups: PTX + S-ketamine + Vehicle, PTX + S-ketamine + CHPG. For all experiments, drugs were administered intraperitoneally (i.p.) at a standardized volume of 10 mL/kg body weight.

**Figure 1:**
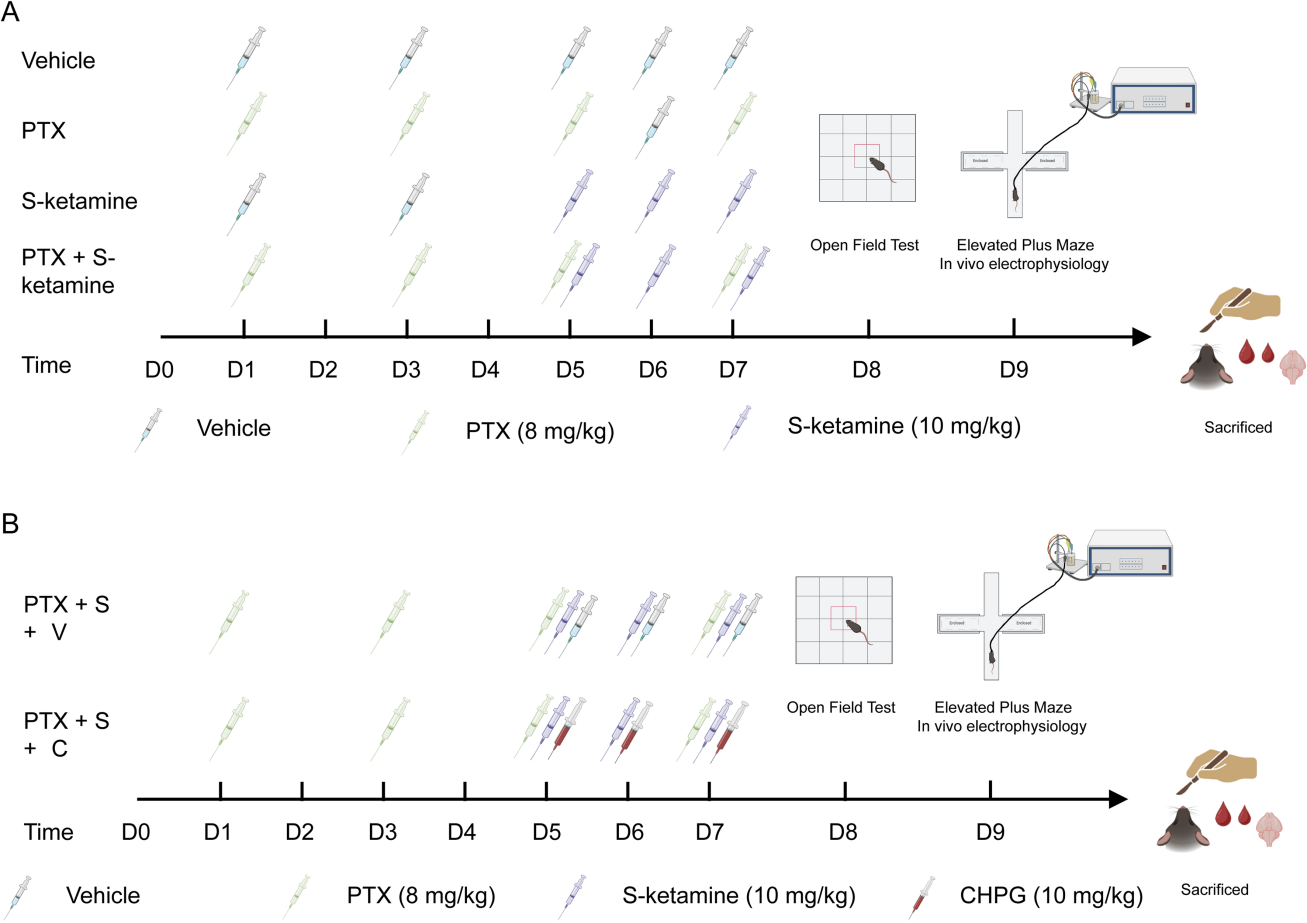
Schematic timelines of the experimental design. **(A)** Timeline for the first set of experiments, illustrating the paclitaxel (PTX)-induced neuropathy modeling and S-ketamine treatment protocol. PTX (8 mg/kg, i.p.) was administered on Days 1, 3, 5, and 7. S-ketamine (10 mg/kg, i.p.) was administered for three consecutive days (Days 5, 6, and 7). Behavioral tests and tissue collection were performed at the indicated time points. **(B)** Timeline for the second set of experiments (mechanistic validation), showing the concurrent administration of S-ketamine and the mGluR5 agonist CHPG. CHPG (10 mg/kg, i.p.) was administered on Days 5, 6, and 7, strictly running parallel to the S-ketamine intervention window (Created with BioRender.com).

PTX (Cat# P1632, TCI, Tokyo, Japan) was dissolved in a mixture of polyoxyethylene castor oil, pure ethanol, and normal saline in a volume ratio of 1:1:13. An intraperitoneal injection of PTX at a dose of 8 mg/kg was administered four times, separated by 1 day, for a total dose of 32 mg/kg. To simulate an early concurrent intervention strategy and ensure sustained synaptic modulation during the active phase of chemotherapy-induced neurotoxicity, the S-ketamine group was injected intraperitoneally with 10 mg/kg S-ketamine (Cat#231021BL, Hengrui Pharmaceutical Co., Ltd., Shanghai, China), which was dissolved in 0.9% normal saline, once daily for three consecutive days (Days 5, 6, and 7), running parallel to the latter half of the PTX cycle. The PTX + S-ketamine group was treated with a combination of PTX and S-ketamine. In the second set of experiments, to investigate whether mGluR5 activation could counteract the therapeutic effects of S-ketamine, the mGluR5 agonist CHPG (Cat#HY-101304; MedChemExpress, New Jersey, United States) also dissolved in 0.9% normal saline, was administered intraperitoneally (i.p.) at a dose of 10 mg/kg once daily for three consecutive days (Days 5, 6, and 7). This timetable was specifically designed to run parallel to the S-ketamine intervention window, ensuring sustained mGluR5 activation during the critical phase of synaptic remodeling. Control mice (Vehicle group) received an equivalent volume of the corresponding vehicle solution to strictly control for solvent-induced effects. The experimental procedure is illustrated in Figure 1. All reagents and equipment used are detailed in Supplementary Document 1.

### 2.3 Mechanical withdrawal threshold

The mechanical withdrawal threshold (MWT) was assessed using an electronic von Frey anesthesiometer (Shanghai Yuyan Instruments Co., Ltd., Shanghai, China). Mice were placed on a metal mesh within a clear glass enclosure (22 cm × 12 cm × 22 cm) and allowed 15 min to acclimate to the environment before testing began. The rigid tip of the device was applied vertically to the center of the left hind paw. A withdrawal response, such as abrupt foot lifting or licking, was considered a positive response, and the applied force (in grams) evoking the withdrawal was recorded automatically. The measurement was repeated three times for each mouse with an inter-trial interval of at least 5 min to avoid sensitization, and the values were averaged to determine the final MWT. To avoid experimental bias, the test was performed by an experimenter who was blinded to the group allocations.

### 2.4 Open field test

To minimize stress-induced confounding factors, a rigorous two-step habituation protocol was strictly followed before behavioral testing. First, the mice, housed in their home cages, were transferred from the main animal holding facility to the behavioral testing room 24 h prior to the start of the behavioral paradigm to acclimate to the general ambient environment. Second, on the actual day of testing, the cages were left completely undisturbed in the testing room for at least 30 min immediately before the experiment commenced. This allowed the mice to calm down after the experimenter’s initial entry and equipment setup. Following this habituation period, the open field test (OFT) was conducted on Day 8 in a quiet and evenly lit independent behavioral laboratory. The open field chamber was a 40 × 40 × 40 cm square opaque plastic box with 16 equal virtual squares (10 × 10 cm) separated on the bottom, and the central area was defined as the four central squares (20 × 20 cm). At the beginning of the experiment, a single mouse was gently placed in the central area of the open field facing the same direction. The experimenter then immediately left the testing room, and the behavior of the mouse was continuously recorded for 5 min using a camera set directly above the box. Human interference was strictly avoided during the recording period. After the test, the mice were quickly returned to their original housing cage. The side walls and bottom of the open field box were completely washed and dried with a 75% ethanol solution to remove any remaining smells before the next mouse was subjected to the test. All operations were performed simultaneously, and the order of mouse testing was randomly arranged (23).

### 2.5 Elevated plus maze

On Day 9 of the experimental timeline, the elevated plus maze (EPM) test was conducted concurrently with *in vivo* electrophysiological recordings. The maze was composed of two opposite open arms (30 × 5 cm, without side walls) and two closed arms (30 × 5 × 15 cm, with high side walls) that intersected vertically. The central platform (5 × 5 cm) connected the four arms, and the entire structure was placed 50 cm above the ground. The experiment was conducted in a quiet and dimly lit environment. A single mouse was gently placed on the central platform with its head facing one of the open arms. During the 5-min testing period, the number of entries into the open arms and the total time spent in the open arms were recorded and analyzed using the Labmaze tracking system (Labmaze V3.0, Beijing Zhongshi Di Chuang Development Technology Co., Ltd., Beijing, China). This analysis was used to evaluate anxiety-like behavior in mice (24).

### 2.6 Immunofluorescence

Six mice per group were selected for immunofluorescence staining after the behavioral research. Following anesthesia with 7% sevoflurane, the mouse hearts were perfused with 10% neutral formalin and pre-cooled normal saline. After complete dissection, the brain tissues were preserved for 24 h at 4 °C in 10% neutral formalin. To ensure anatomical precision, coronal sections (30 μm) containing the prelimbic cortex (PrL) of the mPFC (AP + 1.7 to +2.1 mm from Bregma) obtained by embedding, sectioning, and dehydrating tissues using alcohol gradients. Following a 20-min permeabilization with a 0.3% Triton X-100 solution, the sections were blocked with QuickBlock™ Blocking Buffer for Immunol Staining (Beyotime, Shanghai, China) for 30 min at room temperature to prevent non-specific binding. The sections were then incubated overnight at 4 °C with the following primary antibodies were used (dilution 1:100): Rabbit anti-CaMKII (Cat#ab52476, Abcam), Mouse anti-c-Fos (Cat#ab208942, Abcam), rabbit anti-mGluR5 (Cat#AF1744, Beyotime, Shanghai, China), rabbit anti-BDNF (Cat#AF1423, Beyotime, Shanghai, China), and rabbit anti-PSD95 (Cat#AF1096, Beyotime, Shanghai, China). Following three 10-min phosphate-buffered saline (PBS) washes the following day, the sections were incubated for 1 h in the dark with fluorescently tagged secondary antibodies. The following secondary antibodies were used (dilution 1:500): CyTM3-conjugated goat anti-mouse IgG (Cat#A0521; Beyotime, Shanghai, China) and FITC-conjugated goat anti-rabbit IgG (Cat#A0562; Beyotime, Shanghai, China). The sections were then washed thrice and stained with 4′, 6-diamidino-2-phenylindole for nuclear fixation. A pathologist, who was blinded to the experimental conditions, used a confocal microscope to evaluate the sections. Representative images were captured from layers II/III and V of the PrL. For each group, six regions within the PrL with an amplification factor of ×200 were randomly selected from three sections. The fluorescence intensity of the positive cells was quantitatively examined using ImageJ software.

### 2.7 Western blot

After the behavioral experiment, six mice were anesthetized with 7% sevoflurane and then quickly decapitated to obtain their brains, which were then placed in pre-cooled PBS. The medial prefrontal cortex (mPFC) was precisely dissected on ice according to the mouse brain stereotaxic atlas (Paxinos and Franklin). Specifically, the mPFC region (approximately Bregma +2.0 mm to +1.4 mm) was isolated using micro-dissection tools to ensure anatomical consistency across all samples. After weighing the tissues, RIPA lysis buffer containing protease/phosphatase inhibitors was added, and the samples were sonicated on ice. The protein concentrations were determined using a BCA assay (Beyotime, Shanghai, China) following centrifugation of the lysates at 12,000 × g for 20 min at 4 °C. The final protein concentration was standardized at 5 μg/μL. SDS-PAGE was used to separate 30 μg of protein samples (concentrated gel 5%, separating gel 12%), and then transferred to polyvinylidene fluoride membranes using a wet transfer method. The membrane was blocked with a rapid blocking solution at room temperature for 30 min and then incubated with the primary antibody at 4 °C overnight. The following primary antibodies were used: Polyclonal rabbit anti-mGluR5 (Cat#AF1744, Beyotime, Shanghai, China), polyclonal rabbit anti-BDNF (Cat#AF1423, Beyotime, Shanghai, China), polyclonal rabbit anti-TrkB (Cat#GB11295-1-100, Servicebio, Wuhan, China), polyclonal rabbit anti-phosphorylated TrkB (Cat#AF1963, Beyotime, Shanghai, China), and polyclonal rabbit anti-PSD95 (Cat#AF1096, Beyotime, Shanghai, China). After three 10-min TBST washes the following day, the membranes were incubated for 1 h at room temperature with an HRP-labeled secondary antibody. The membranes were developed using ECL chemiluminescent reagents, and the gray values of the bands were analyzed using ImageJ software. GAPDH served as an internal reference to normalize the expression of target proteins.

### 2.8 *In vivo* electrophysiological recording

To investigate the neuronal oscillation mechanisms within the prelimbic cortex (PrL) subregion of medial prefrontal cortex (mPFC) underlying anxiety-like behaviors, *in vivo* electrophysiological recordings were performed. The mice (*n* = 3 per group) were randomly grouped by a computer, anesthetized with sevoflurane (7% induction and 2% maintenance), and fixed on a stereotactic instrument. According to the Paxinos and Franklin mouse brain atlas, a 16-channel silicon probe microelectrode array (Jiangsu Brain Medical Technology Co., Ltd., Nanjing, China) was precisely implanted into the PrL. The stereotactic coordinates were as follows: AP + 1.9 mm, ML 0.35 mm, and DV 1.7–2.2 mm from the dural surface. This range was specifically optimized to target the pyramidal cell layers (Layers II/III and V) of the PrL. After sealing the cranial window with dental cement, following the procedure, mice were individually housed, with one animal per cage, and allowed to recover for 1 week to ensure the integrity of the implanted electrodes. During the EPM behavioral test, local field potentials and single-unit spike activities of putative pyramidal neurons were synchronously collected using a wireless transmitter. Moreover, the anxiety-related behaviors of the mice, including the time spent in the open arms and the number of open-arm entries, were recorded using a high-definition camera and analyzed to assess their anxiety levels. The raw signals were amplified and analyzed offline. An FIR filter band-pass filter was employed to separate the theta (4–8 Hz) and gamma (30–80 Hz) bands. A 5-s window was extracted before and after the ‘event,’ which was defined as the exact moment the mouse entered the open arms (all four paws across the threshold). The Power Spectral Density (PSD) was calculated using Welch’s periodogram method. To account for the 1/f nature of the LFP signal and ensure the data met the assumptions of normality for parametric statistical analysis, raw power values were log-transformed and expressed in decibels (dB) relative to the baseline period [10 × log_10_ (Power_event_/Power_baseline_)]. This normalization of PSD (dB) stabilizes the variance and reduces the skewness inherent in raw power distributions, allowing for a more reliable statistical comparison of power changes across different frequency oscillations. Phase-amplitude coupling (PAC) is a prevalent type of cross-frequency coupling (25). This phenomenon explains the connection between the amplitude of the gamma oscillations and the phase of the low-frequency rhythms (theta). The strength of this coupling was quantified using the Modulation Index (MI), which measures the extent to which the gamma amplitude is distributed across the theta phases. Establishing a link between theta and gamma coupling and behavioral changes may offer therapeutic insights (26). The MI was used to evaluate the modulation strength of the theta phase on the gamma amplitude in relation to the mice’s behavioral alterations.

### 2.9 Statistical analysis

All experimental procedures, including behavioral testing, histological quantification, and offline electrophysiological analyses, were performed by researchers who were strictly blinded to the experimental group assignments. Throughout all statistical analyses, “*n*” explicitly represents the number of independent animals (biological replicates), rather than the number of sections or cells. For histological (immunofluorescence and Western blot) and electrophysiological assessments, multiple measurements obtained from a single animal (e.g., at least 3 non-adjacent brain sections) were averaged to generate a single independent data point per animal prior to statistical comparison. All statistical analyses were conducted using GraphPad Prism software (version 9.5.0; GraphPad Software, San Diego, CA, United States). Data distribution was first evaluated using normality and log-normality tests (e.g., Shapiro–Wilk test). For normally distributed data with a single independent variable (such as OFT, EPM, IF, and WB data), a one-way analysis of variance (ANOVA) was utilized. For data involving multiple time points (specifically, the mechanical withdrawal threshold [MWT] measurements over the 8-day period), a two-way repeated-measures ANOVA was employed to assess the main effects of time and treatment, as well as their interaction. Where ANOVA indicated significant main effects or interactions, Tukey’s *post hoc* multiple comparison test was applied to determine specific group differences. All data are presented as the mean ± standard deviation (SD). A *p* < 0.05 was considered statistically significant. The detailed statistical findings are presented in Supplementary File 2.

## 3 Results

### 3.1 S-ketamine reversed PTX-induced mechanical allodynia and anxiety-like behaviors

**Figure 2:**
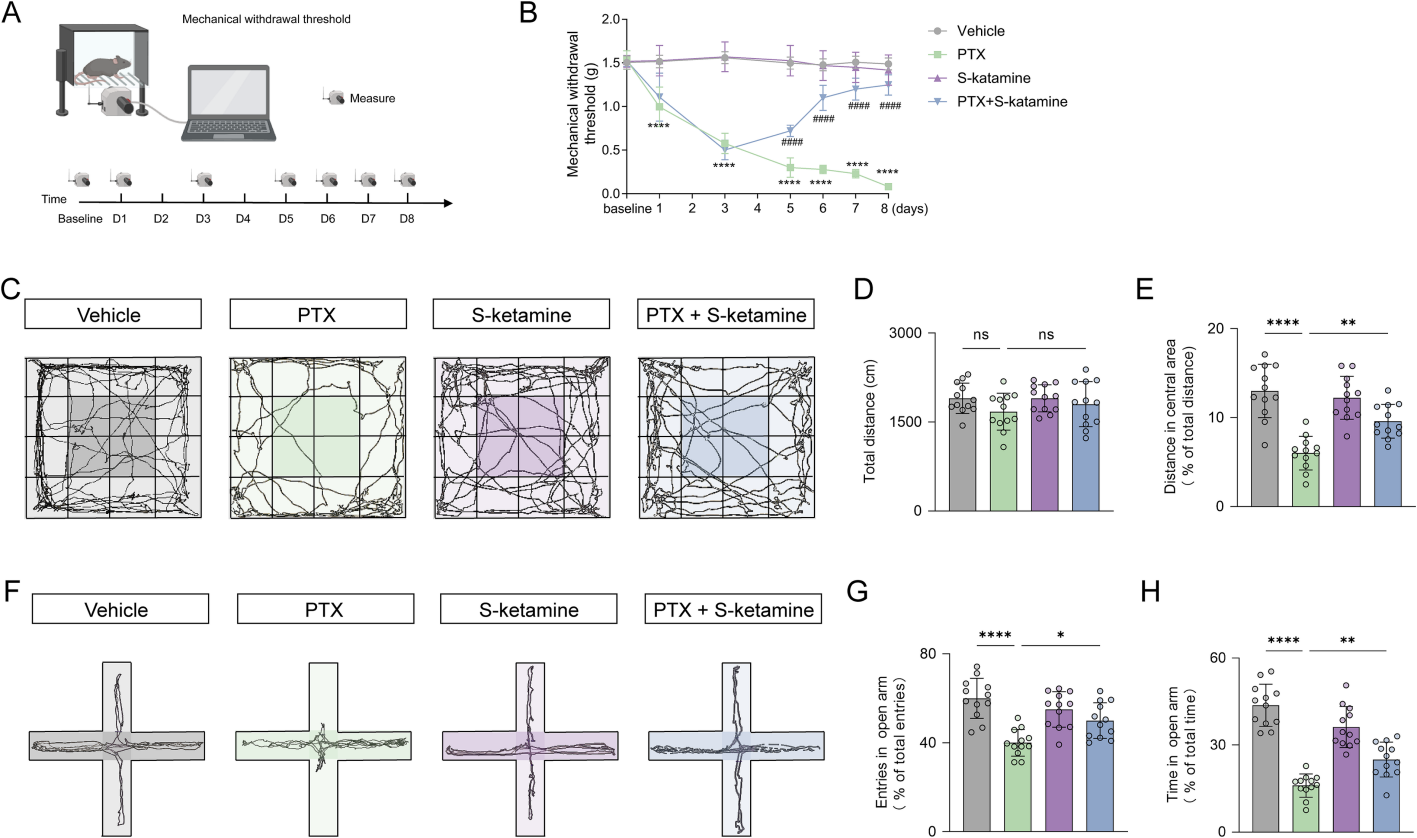
S-ketamine reverses PTX-induced mechanical allodynia and anxiety-like behaviors. **(A)** Schematic diagram of the experimental setup and timeline for testing the mechanical withdrawal threshold. **(B)** Time course of the mechanical withdrawal threshold (MWT) measured by the electronic von Frey test over an 8-day period. **(C)** Representative movement trajectories of mice in the open field test (OFT). **(D,E)** Quantitative analysis of the total distance traveled **(D)** and the percentage of distance traveled in the central area **(E)** in the OFT. **(F)** Representative movement trajectories of mice in the elevated plus maze (EPM) test. **(G,H)** Quantitative analysis of the percentage of entries into the open arms **(G)** and the percentage of time spent in the open arms **(H)** in the EPM. Data are presented as mean ± SD. *n* = 12 mice per group. Statistical significance was determined by two-way repeated-measures ANOVA for the MWT data (B) and one-way ANOVA for the remaining quantitative behavioral data **(D,E,G,H)**, followed by Tukey’s *post hoc* test. *p < 0.05, *p* < 0.01, *p < 0.001, ****p < 0.0001. PTX, paclitaxel. In all experiments, “n” represents the number of independent animals (biological replicates). For histological and electrophysiological assessments, data were averaged per animal prior to statistical comparisons. All behavioral testing, histological quantification, and offline data analyses were conducted under blinded conditions.

To validate the establishment of the paclitaxel (PTX)-induced peripheral neuropathy model and evaluate the analgesic effect of S-ketamine, we first assessed the mechanical withdrawal threshold (MWT) of the mice (Figures 2A,B). As shown in Figure 2B, repeated PTX administration significantly decreased the MWT starting from day 1 compared to the vehicle group, indicating the successful induction of severe mechanical allodynia. Conversely, continuous S-ketamine treatment significantly attenuated this PTX-induced mechanical hypersensitivity, with robust and significant recovery observed from day 5 onwards.

Following the completion of the administration, open field test (OFT) and elevated plus maze (EPM) trials were conducted to assess anxiety-like behaviors. In the OFT experiment (Figure 2C), compared with the PTX group, the PTX + S-ketamine group exhibited no significant difference in the total distance (Figure 2D); however, the percentage of distance traveled in the central area was significantly increased (Figure 2E). In the EPM experiment (Figure 2F), compared with the PTX group, the PTX + S-ketamine group exhibited a significant increase in the percentage of open-arm entry (Figure 2G) and the percentage of time spent in the open arms (Figure 2H). These behavioral results collectively demonstrate that S-ketamine effectively alleviates PTX-induced neuropathic pain and its concurrent anxiety-like behaviors.

### 3.2 S-ketamine restored the electrophysiological properties and neural oscillations in the mPFC

**Figure 3:**
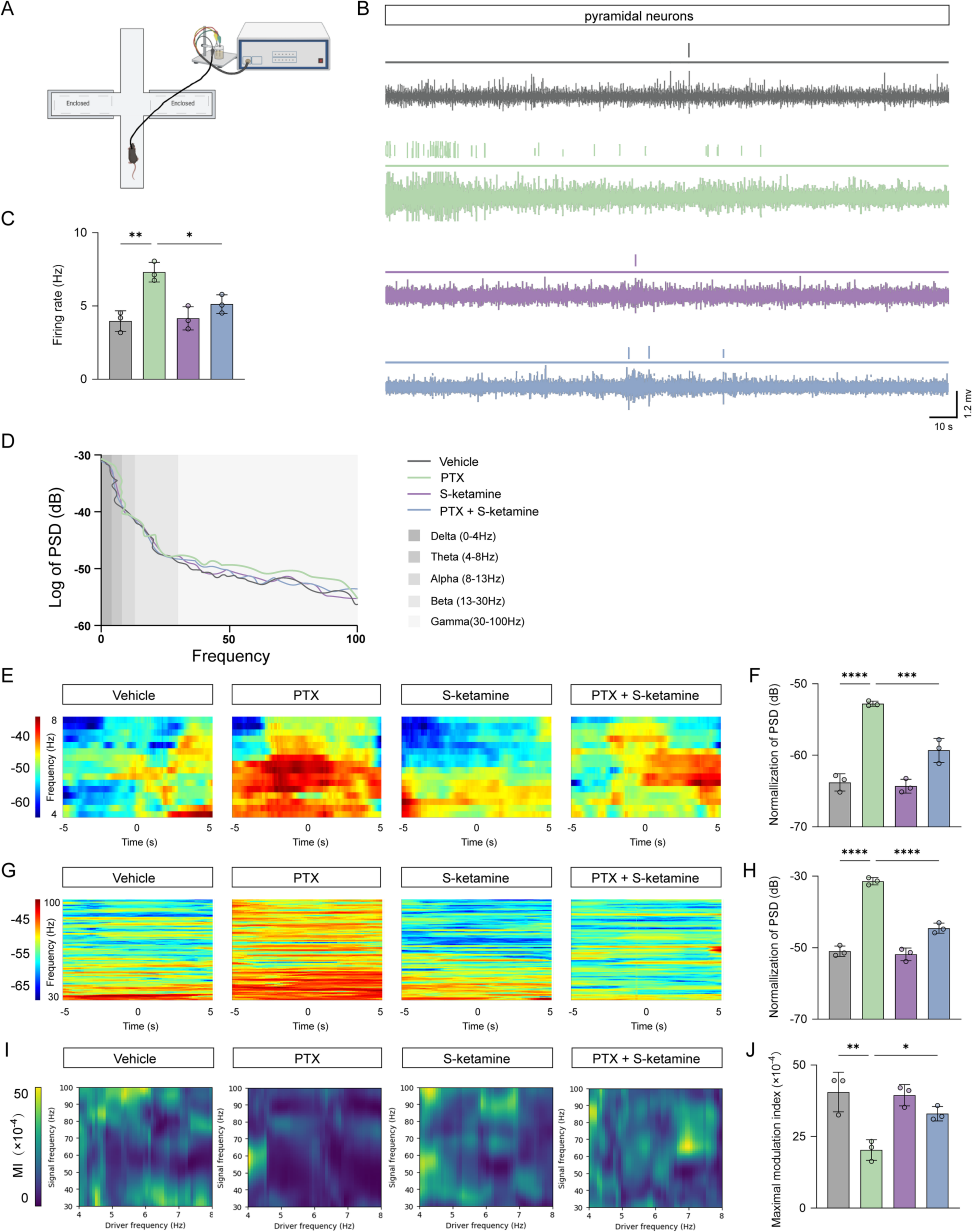
S-ketamine restores neuronal firing properties and rhythmic oscillations in the PrL of the mPFC. **(A)** Schematic diagram of *in vivo* multi-channel electrophysiological recording in the prelimbic cortex (PrL) subregion of the mPFC during the elevated plus maze (EPM) test. **(B)** Representative raw traces and filtered spike waveforms of putative pyramidal neurons across the four experimental groups. **(C)** Quantitative analysis of the spontaneous firing rate (Hz) of putative pyramidal neurons. **(D)** Grand average of the power spectral density (PSD) from 0 to 100 Hz in the PrL. **(E,F)** Representative time-frequency spectrograms **(E)** and normalized power statistics **(F)** for theta (4–8 Hz) oscillations. **(G,H)** Representative time-frequency spectrograms **(G)** and normalized power statistics **(H)** for gamma (30–100 Hz) oscillations. **(I,J)** Cross-frequency coupling analysis. Representative pseudocolor maps of theta-gamma phase-amplitude coupling (PAC) **(I)** and quantitative analysis of the maximal modulation index (MI) **(J)**. Data are presented as mean ± SD. n = 3 mice per group. Statistical significance was determined by one-way ANOVA followed by Tukey’s *post hoc* test. *p < 0.05, **p < 0.01, ***p < 0.001, ****p < 0.0001. mPFC, medial prefrontal cortex; PSD, power spectral density; MI, modulation index. In all experiments, “n” represents the number of independent animals (biological replicates). For histological and electrophysiological assessments, data were averaged per animal prior to statistical comparisons. All behavioral testing, histological quantification, and offline data analyses were conducted under blinded conditions.

To further investigate the network-level mechanisms underlying the behavioral rescue, we performed *in vivo* multi-channel electrophysiological recordings specifically within the prelimbic cortex (PrL) subregion of the mPFC (targeted at layers II/III and V) while mice were undergoing the EPM test (Figure 3A). Representative traces of neuronal discharge activities are shown in Figure 3B. Based on established electrophysiological criteria, the recorded units were classified as putative pyramidal (predominantly glutamatergic) neurons. Compared to the vehicle group, PTX significantly increased the spontaneous discharge frequency of these neurons in the PrL. Conversely, S-ketamine treatment successfully attenuated this hyperactive firing (Figure 3C).

Beyond single-unit activity, we analyzed local field potentials (LFPs) to assess macroscopic neural network oscillations (Figure 3D). Time-frequency spectrograms revealed that PTX administration induced an abnormal increase in neural oscillatory power, particularly within the gamma band (Figures 3E,G). Statistical analysis of the normalized power spectral density (PSD) confirmed that continuous S-ketamine treatment significantly suppressed this PTX-induced aberrant gamma hyperactivity (Figures 3F,H).

Cross-frequency coupling, specifically theta-gamma phase-amplitude coupling (PAC), is crucial for coordinating PrL mPFC local circuit activity and emotional regulation. We calculated the modulation index (MI) to quantify this dynamic interaction (Figure 3I). The results demonstrated that PTX severely disrupted theta-gamma coupling, whereas S-ketamine treatment significantly reinstated the impaired cross-frequency coupling (Figure 3J). Collectively, these *in vivo* recordings suggest that S-ketamine mitigates anxiety-like behaviors by re-establishing the excitatory/inhibitory balance and restoring coordinated neural network dynamics in the mPFC.

### 3.3 S-ketamine attenuated the overactivation of CaMKII-positive pyramidal neurons, downregulated mGluR5, and restored the BDNF/TrkB signaling pathway in the mPFC

**Figure 4:**
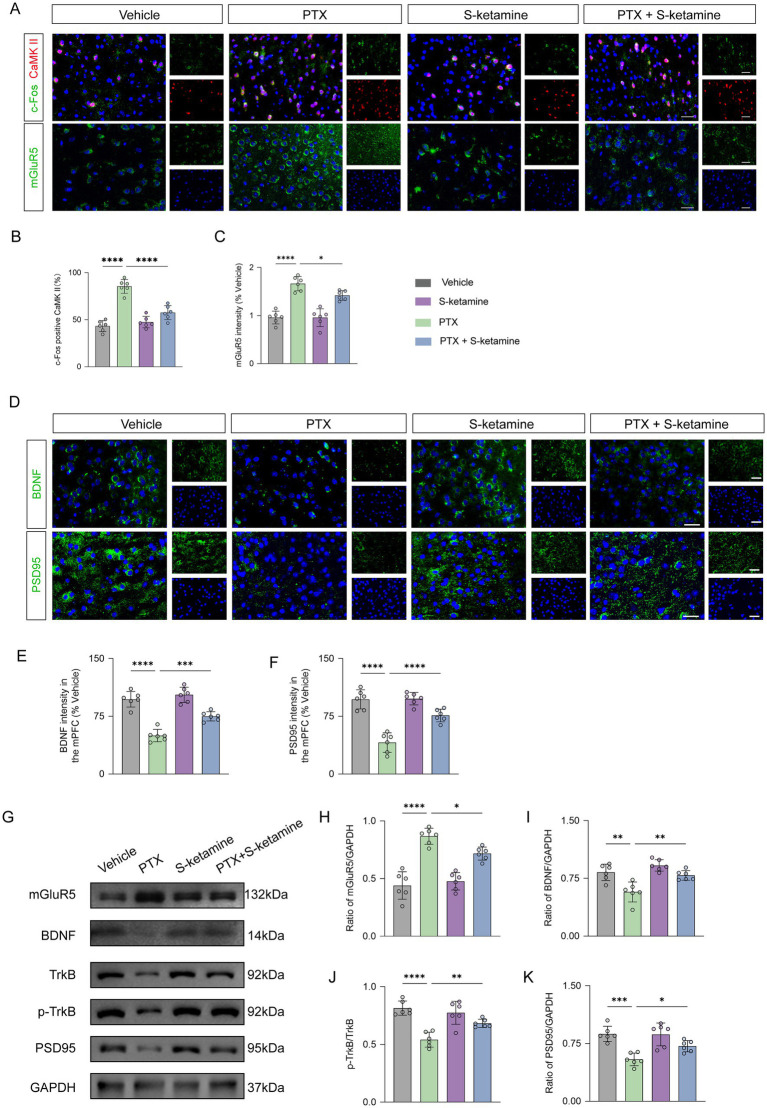
S-ketamine suppresses mGluR5-mediated pyramidal neuron hyperactivity and rectifies the BDNF/TrkB signaling pathway in the PrL of the mPFC. **(A–C)** Immunofluorescence analysis of neuronal activation and mGluR5 expression. Representative images **(A)** and quantitative analysis of the percentage of c-Fos-positive CaMKII cells **(B)** and mGluR5 fluorescence intensity **(C)** in the mPFC. Scale bar = 50 μm (insert: 20 μm). **(D–F)** Morphological assessment of synaptic and neurotrophic markers. Representative immunofluorescence images **(D)** and quantitative analysis of BDNF **(E)** and PSD95 **(F)** fluorescence intensities in the mPFC. Scale bar = 50 μm (insert: 20 μm). **(G)** Representative Western blot bands showing the protein expression levels of mGluR5, BDNF, TrkB, p-TrkB, and PSD95, with GAPDH as the internal control. **(H–K)** Densitometric quantification of Western blot results. Relative protein levels of mGluR5/GAPDH **(H)**, BDNF/GAPDH **(I)**, p-TrkB/TrkB ratio **(J)**, and PSD95/GAPDH **(K)**. Data are presented as mean ± SD. *n* = 6 mice per group for immunofluorescence **(A–F)**; *n* = 6 mice per group for Western blot **(G–K)**. Statistical significance was determined by one-way ANOVA followed by Tukey’s *post hoc* test. *p < 0.05, **p < 0.01, ***p < 0.001, ****p < 0.0001. mPFC, medial prefrontal cortex; CaMKII, calcium/calmodulin-dependent protein kinase II; BDNF, brain-derived neurotrophic factor; PSD95, postsynaptic density protein 95. In all experiments, “n” represents the number of independent animals (biological replicates). For histological and electrophysiological assessments, data were averaged per animal prior to statistical comparisons. All behavioral testing, histological quantification, and offline data analyses were conducted under blinded conditions.

To investigate the underlying cellular and molecular mechanisms of PTX-induced affective comorbidities and the therapeutic effects of S-ketamine, we assessed neuronal activation markers and key signaling proteins in the PrL subregion of mPFC. c-Fos is an immediate early gene (IEG), representing an immediate marker of neuronal activity. Calcium/calmodulin-dependent protein kinase II (CaMKII) is a specific and widely recognized marker for excitatory pyramidal neurons. Our immunofluorescence findings indicated that, compared with the vehicle group, PTX significantly increased neuronal activation. Conversely, continuous S-ketamine treatment significantly reduced the percentage of c-Fos-positive CaMKII cells (which represent activated pyramidal neurons) within layers II/III and V of the PrL (AP + 1.7 to +2.1 mm)(Figures 4A,B). Furthermore, immunofluorescence staining revealed that PTX induced a pronounced overexpression of mGluR5, whereas S-ketamine simultaneously downregulated this expression (Figures 4A,C).

Synaptic plasticity and neurotrophic support are deeply implicated in neuropathic pain and anxiety-like behaviors. Therefore, we further investigated the brain-derived neurotrophic factor (BDNF) signaling pathway and the postsynaptic density protein PSD95. Immunofluorescence staining demonstrated that PTX treatment significantly reduced the fluorescence intensities of BDNF and PSD95 in the mPFC, which were effectively rescued by S-ketamine administration (Figures 4D–F).

These morphological changes were robustly corroborated by Western blot analyses (Figure 4G). Quantitative results showed that S-ketamine significantly reversed the PTX-induced upregulation of mGluR5 protein expression (Figure 4H) and the profound downregulation of BDNF (Figure 4I). Moreover, S-ketamine effectively restored the phosphorylation level of its high-affinity receptor TrkB (represented by the p-TrkB/TrkB ratio, Figure 4J) and the expression of the synaptic marker PSD95 (Figure 4K). Collectively, these molecular changes strongly align with the *in vivo* electrophysiological findings, indicating that S-ketamine exerts its therapeutic effects by suppressing mGluR5-mediated pyramidal neuron hyperactivity and rectifying deficits in neurotrophic signaling.

### 3.4 Activation of mGluR5 by CHPG abolished the behavioral and electrophysiological rescue effects of S-ketamine

**Figure 5:**
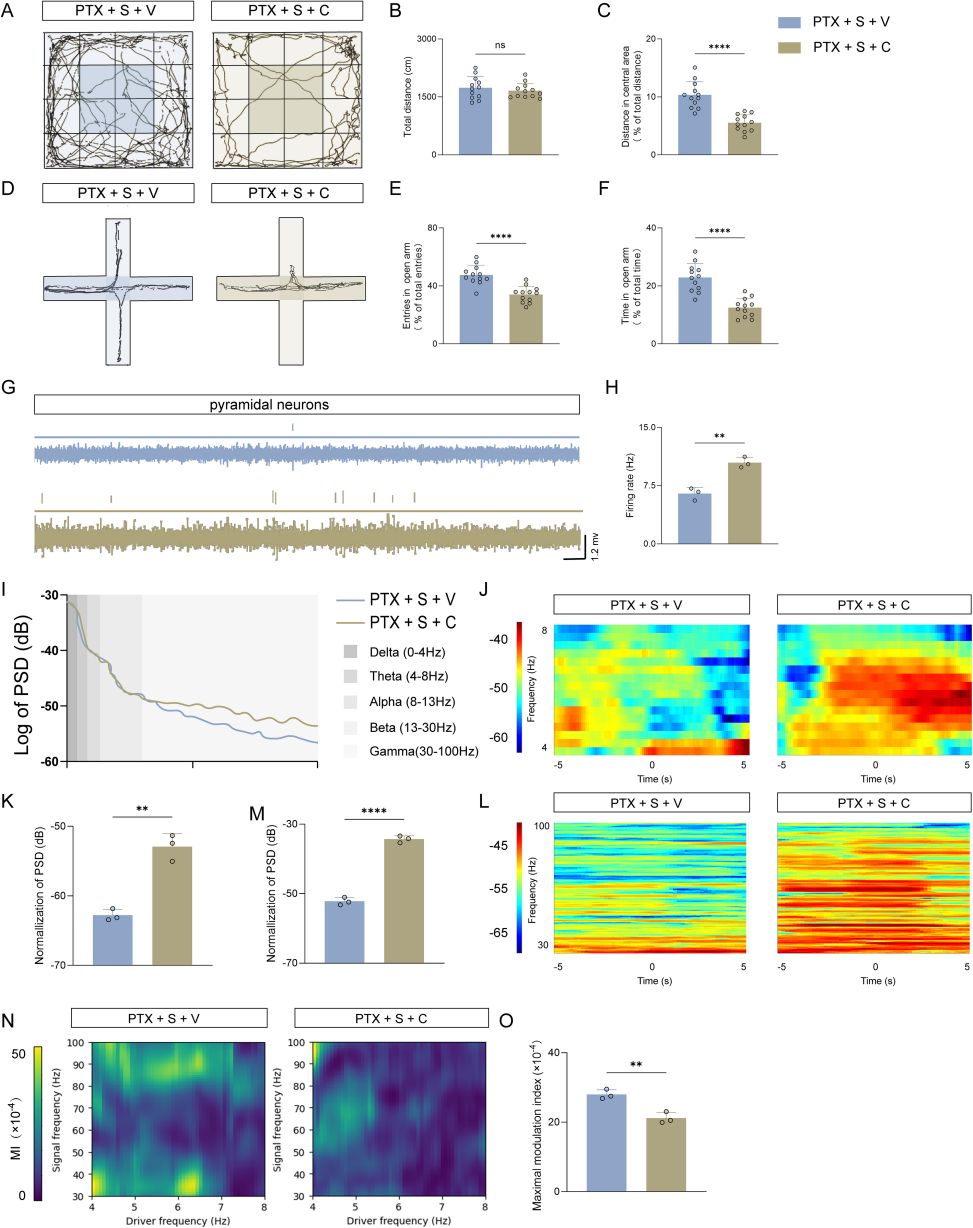
Activation of mGluR5 by CHPG abolishes the behavioral and electrophysiological rescue effects of S-ketamine. **(A–C)** Behavioral assessment in the open field test (OFT). Representative movement trajectories **(A)**, and quantitative analysis of total distance **(B)** and percentage of distance traveled in the central area **(C)**. **(D–F)** Behavioral assessment in the elevated plus maze (EPM) test. Representative movement trajectories **(D)**, and quantitative analysis of the percentage of open-arm entries **(E)** and the percentage of time spent in the open arms **(F)**. **(G,H)** *In vivo* single-unit recording in the mPFC. Representative discharge traces **(G)** and statistical analysis of the spontaneous firing rate **(H)** of putative pyramidal neurons. **(I–M)** Local field potential (LFP) oscillation analysis. Grand average of the power spectral density (PSD) **(I)**, representative time-frequency spectrograms **(J,L)**, and normalized power statistics for theta (4–8 Hz) **(K)** and gamma (30–100 Hz) **(M)** oscillations. **(N,O)** Cross-frequency coupling analysis. Representative pseudocolor maps of theta-gamma phase-amplitude coupling (PAC) **(N)** and quantitative analysis of the maximal modulation index (MI) **(O)**. Data are presented as mean ± SD. *n* = 12 mice per group for behavioral tests **(A–F)**; *n* = 3 mice per group for electrophysiological recordings **(G–O)**. Statistical significance was determined by two-tailed unpaired t-test. *p < 0.05, **p < 0.01, ***p < 0.001, ****p < 0.0001; ns, not significant. PTX, paclitaxel; S, S-ketamine; V, vehicle; C, CHPG. In all experiments, “*n*” represents the number of independent animals (biological replicates). For histological and electrophysiological assessments, data were averaged per animal prior to statistical comparisons. All behavioral testing, histological quantification, and offline data analyses were conducted under blinded conditions.

To causally confirm whether the therapeutic effects of S-ketamine are mediated by the inhibition of mGluR5, we performed a pharmacological rescue experiment using the selective mGluR5 agonist, CHPG. Mice were treated with CHPG (PTX + S + C) or a vehicle control (PTX + S + V) following S-ketamine administration to determine if re-activating mGluR5 could counteract the observed improvements. In the open field test (OFT), while the total distance remained unchanged between the two groups (Figures 5A,B), CHPG administration significantly reduced the percentage of distance traveled in the central area (Figure 5C). Similarly, in the elevated plus maze (EPM) test, re-activating mGluR5 significantly decreased both the percentage of open-arm entries (Figures 5D,E) and the time spent in the open arms (Figure 5F). These behavioral results indicate that CHPG effectively reversed the anxiolytic effects of S-ketamine.

We further investigated whether mGluR5 activation disrupted the S-ketamine-mediated restoration of neural circuit function. *In vivo* multi-channel recordings revealed that CHPG administration significantly increased the spontaneous discharge frequency of putative pyramidal neurons in the PrL, re-inducing a hyperactive firing state (Figures 5G,H). Furthermore, local field potential (LFP) analysis demonstrated that CHPG reversed the network normalization, leading to a significant increase in neural oscillation power (Figures 5I–M) and a profound weakening of the theta-gamma phase-amplitude coupling (PAC) (Figures 5N,O). Collectively, these *in vivo* findings provide compelling evidence that S-ketamine mitigates PTX-induced anxiety-like behaviors specifically by suppressing mGluR5-mediated pyramidal neuron overactivation and rectifying neural network dysregulation.

### 3.5 Activation of mGluR5 by CHPG abolished the S-ketamine-mediated molecular rescue and suppressed the BDNF/TrkB signaling pathway

**Figure 6:**
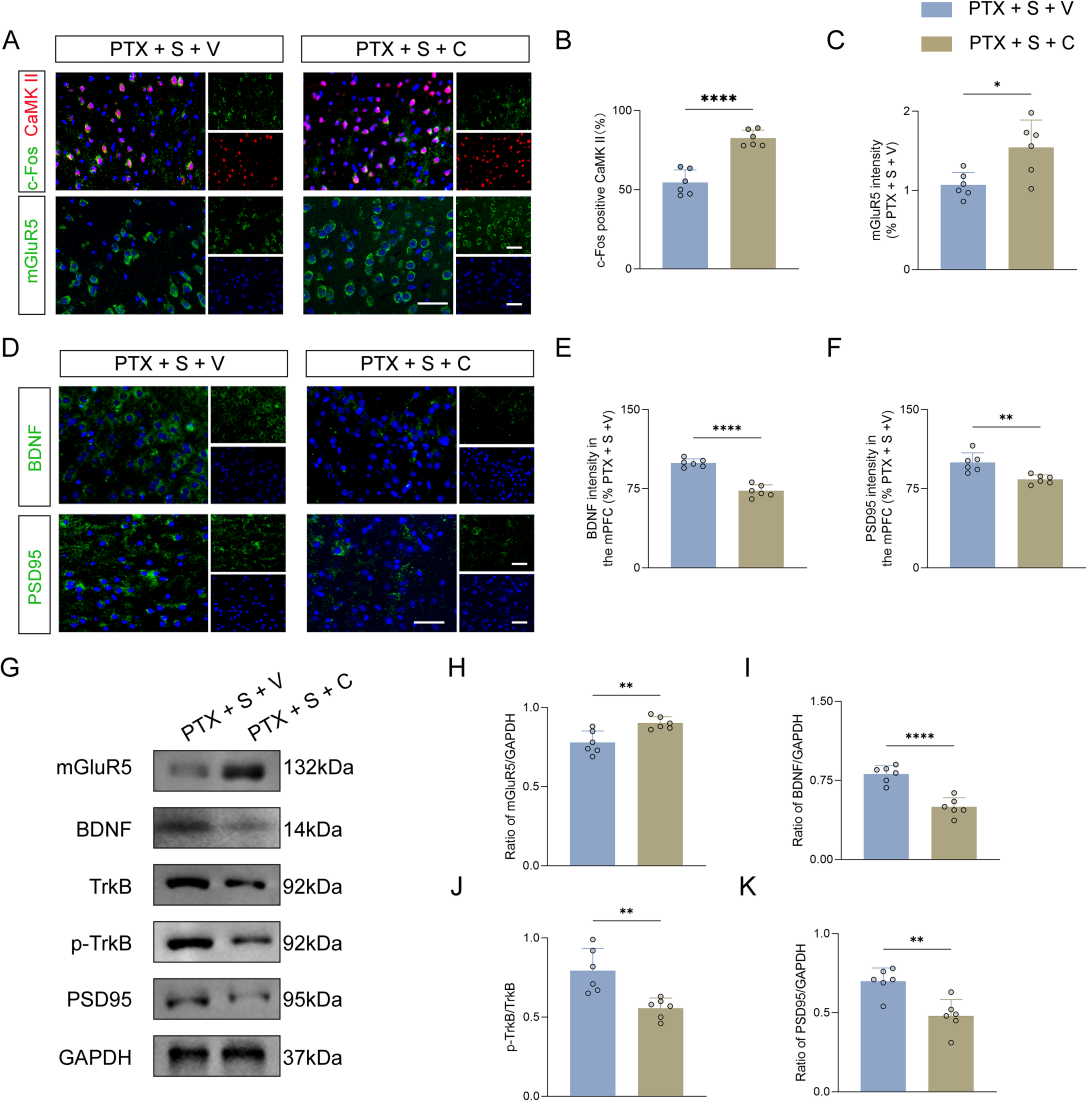
Activation of mGluR5 by CHPG abolishes the S-ketamine-mediated molecular rescue and suppresses the BDNF/TrkB signaling pathway. **(A–C)** Immunofluorescence analysis of pyramidal neuron activation and mGluR5 expression. Representative images **(A)** and quantitative analysis of the percentage of c-Fos-positive CaMKII cells **(B)** and mGluR5 fluorescence intensity **(C)** in the mPFC. Scale bar = 50 μm (insert: 20 μm). **(D–F)** Morphological assessment of neurotrophic and synaptic markers. Representative immunofluorescence images **(D)** and quantitative analysis of BDNF **(E)** and PSD95 **(F)** fluorescence intensities in the mPFC. Scale bar = 50 μm (insert: 20 μm). **(G)** Representative Western blot bands showing the protein expression levels of mGluR5, BDNF, TrkB, p-TrkB, and PSD95, with GAPDH as the internal control. **(H–K)** Densitometric quantification of Western blot results. Relative protein levels of mGluR5/GAPDH **(H)**, BDNF/GAPDH **(I)**, p-TrkB/TrkB ratio **(J)**, and PSD95/GAPDH **(K)**. Data are presented as mean ± SD. *n* = 6 mice per group. Statistical significance was determined by two-tailed unpaired *t*-test. *p < 0.05, **p < 0.01, ***p < 0.001, ****p < 0.0001. PTX, paclitaxel; S, S-ketamine; V, vehicle; C, CHPG; CaMKII, calcium/calmodulin-dependent protein kinase II; BDNF, brain-derived neurotrophic factor; PSD95, postsynaptic density protein 95. In all experiments, “n” represents the number of independent animals (biological replicates). For histological and electrophysiological assessments, data were averaged per animal prior to statistical comparisons. All behavioral testing, histological quantification, and offline data analyses were conducted under blinded conditions.

To determine whether the behavioral and electrophysiological reversals induced by CHPG were accompanied by corresponding molecular alterations, we evaluated the cellular activation and neurotrophic signaling in the mPFC. Consistent with the *in vivo* hyperactive firing data, immunofluorescence analysis revealed that re-activating mGluR5 with CHPG (the PTX + S + C group) significantly increased the percentage of c-Fos-positive CaMKII cells compared to the vehicle control (the PTX + S + V group), indicating a re-emergent overactivation of excitatory pyramidal neurons (Figures 6A,B). Concurrently, CHPG administration significantly upregulated the fluorescence intensity of mGluR5 (Figures 6A,C). Furthermore, the S-ketamine-induced restoration of synaptic plasticity markers was completely blocked by CHPG, as evidenced by a profound reduction in the fluorescence intensities of BDNF and PSD95 in the PrL (Figures 6D–F).

These morphological observations were robustly supported by Western blot analyses (Figure 6G). Quantitative data demonstrated that CHPG treatment significantly increased mGluR5 protein expression (Figure 6H) while drastically decreasing the expression of BDNF (Figure 6I). Moreover, CHPG administration significantly attenuated the phosphorylation of TrkB (indicated by a reduced p-TrkB/TrkB ratio, Figure 6J) and downregulated the synaptic marker PSD95 (Figure 6K). Collectively, these molecular findings form a highly consistent mechanistic link with our behavioral and electrophysiological data, strongly suggesting that S-ketamine exerts its dual analgesic and anxiolytic effects by inhibiting mGluR5-mediated pyramidal neuron overactivation and subsequently rescuing BDNF/TrkB-dependent synaptic plasticity in the PrL.

## 4 Discussion

In the present study, we investigated the therapeutic potential and underlying mechanisms of S-ketamine in a mouse model of paclitaxel (PTX)-induced neuropathic pain and related anxiety. Our results logically demonstrated a clear pathological cascade: PTX induced severe mechanical allodynia and anxiety-like behaviors, which were accompanied by aberrant local neural network oscillations, mGluR5 overactivation, and impaired BDNF/TrkB signaling in the prelimbic cortex (PrL). Administration of S-ketamine robustly alleviated these behavioral and electrophysiological deficits, while simultaneously downregulating mGluR5 and rescuing the BDNF/TrkB pathway. Furthermore, pharmacological re-activation of mGluR5 using CHPG abolished the therapeutic effects of S-ketamine, providing strong pharmacological evidence for the critical role of the mGluR5-BDNF/TrkB axis in mediating these pain-induced affective deficits. Chemotherapy-induced pain usually progresses to chronic neuropathic pain as PTX dosage and treatment duration rise. This maladaptive sensory state is frequently accompanied by affective disorders (27). Consistent with this clinical progression, the PTX-treated mice in our study developed severe mechanical allodynia, as evidenced by significantly reduced paw withdrawal thresholds. Following the emergence of this pain phenotype, the PTX-treated mice exhibited fewer entries and shorter durations in the open arms and the central area of the EPM and OFT, respectively, demonstrating robust pain-related anxiety-like behavior.

To understand the network-level basis of these behavioral changes, we evaluated local field potentials in the PrL. Neural oscillations serve not only as fundamental mechanisms for coordinating local neural assemblies but also facilitate long-range communication through synchronized activity across distributed brain regions (28). Aberrant oscillatory patterns are recognized as crucial pathophysiological features of anxiety-like behavior. In rodent models, elevated PSD (log-transformed dB) in both the gamma (30–100 Hz) and theta (4–8 Hz) frequency regions is reliably associated with symptoms of anxiety (29). mPFC neurons exhibiting theta entrainment demonstrate heightened activity modulation during investigation of the EPM’s open arms (29). Consistent with this, our experimental data revealed that PTX-treated mice displayed abnormally increased theta and gamma power specifically during open arm exploration, aligning with established pathological signatures. Furthermore, phase-amplitude coupling (PAC) characterizes a hierarchical interaction wherein the amplitude of high-frequency oscillations is modulated by the phase of low-frequency oscillations (30). Gamma oscillations emerge from precisely timed connections between inhibitory interneurons and excitatory pyramidal cells within local circuits (31). Given its role in coordinating neural information transfer, disrupted theta-gamma PAC represents a promising therapeutic target for anxiety disorders. In our study, within the 5 s before and after the mice entered the open arm, theta-gamma PAC was significantly disrupted in PTX-treated mice. However, administration of S-ketamine effectively suppressed the aberrant hyperactive oscillations and reinstated the impaired cross-frequency coupling, which perfectly corresponded to the behavioral improvements.

At the cellular and molecular levels, PTX acts on the PrL subregion of the mPFC to elevate intracellular calcium concentrations, activate NMDA receptors (NMDARs), and drive neuropathic pain-associated emotional disturbances (32, 33). Ketamine, an enantiomeric mixture containing R-ketamine and S-ketamine, exerts its effects partially through NMDAR modulation, with S-ketamine demonstrating a fourfold greater NMDAR affinity than R-ketamine (34, 35). It is well established that ketamine preferentially blocks NMDA receptors on GABAergic interneurons, leading to the disinhibition of pyramidal neurons and an acute glutamate surge. This initial surge is a critical catalyst for use-dependent synaptic plasticity, notably triggering the activation of the BDNF/TrkB signaling pathway (36, 37). In our PTX-induced model, where putative pyramidal neurons are pathologically hyperactive, we propose that this S-ketamine-triggered rapid synaptic remodeling, working in concert with the targeted downregulation of mGluR5, ultimately ‘resets’ the aberrant local circuit. Our results demonstrated that S-ketamine reverses PTX-induced anxiety-like behaviors by downregulating mGluR5 overexpression and attenuating excessive pyramidal neuron activation. It is noteworthy that while S-ketamine significantly downregulates mGluR5 expression, it does not completely restore it to absolute control levels. Despite this partial molecular restoration, the mice displayed a robust recovery in anxiety-like behaviors. This apparent discrepancy suggests that neural circuits governing complex emotional behaviors likely operate on threshold dynamics rather than a strict linear correlation with single-protein levels. Attenuating mGluR5 below a critical pathological threshold, combined with the concurrent robust activation of the BDNF/TrkB pathway, appears functionally sufficient to re-establish the overall excitatory/inhibitory (E/I) balance and normalize network oscillations, thereby achieving a comprehensive behavioral rescue (38).

The subsequent activation of the neurotrophic cascade is vital for sustaining this rescue. BDNF is a crucial component of chronic pain disorders, and alterations in its function contribute to various neuropsychiatric syndromes, including anxiety disorders (39). BDNF, released from presynaptic terminals or dendrites, modulates neuronal growth, differentiation, and synaptic plasticity through TrkB activation (40). Within emotion-regulating brain regions, including the PrL, the BDNF–TrkB signaling pathway critically mediates the cellular and molecular mechanisms underlying neuronal circuit remodeling (41). Studies utilizing a social defeat stress model of depression demonstrated that reduced BDNF protein expression in the PrL is associated with attenuated BDNF–TrkB signaling, decreased dendritic spine density, synaptic loss, and the emergence of depressive-like behaviors (42). mGluR5 is functionally interconnected with BDNF–TrkB signaling and is implicated in depression-like behaviors (43). Our pharmacological rescue experiment demonstrates that, in a PTX-induced paradigm, re-activating mGluR5 with CHPG abolished the S-ketamine-mediated restoration of BDNF/TrkB signaling. This further supports the notion that S-ketamine reduces anxiety-like behaviors by simultaneously downregulating the expression of mGluR5 and subsequently rescuing the BDNF–TrkB signaling pathway.

This study has several limitations that warrant consideration. First, our experiments were conducted exclusively using adult male, non-tumor-bearing mice. Clinically, paclitaxel is administered to oncology patients—both male and female—who present with complex interacting variables, including tumor-induced systemic inflammation, disease-related morbidities, and profound psychological burdens. Furthermore, pain and affective disorders frequently exhibit significant sex dimorphism. Therefore, the absence of female subjects and tumor-bearing models limits the direct clinical generalizability of our findings, necessitating future studies that incorporate these crucial clinical variables. Second, while our *in vivo* electrophysiological recordings yielded consistent and statistically significant differences, the sample size for these specific experiments was relatively small (*n* = 3 per group). Consequently, while our pharmacological interventions strongly suggest a functional link between the mGluR5-BDNF/TrkB axis and PrL oscillatory dynamics, the precise causal sequence and direct mechanistic coupling warrant a more cautious interpretation and validation in larger cohorts. Finally, our anatomical focus was restricted to the PrL subregion of the mPFC; other emotion- and pain-related regions, such as the hippocampus, amygdala, and striatum, require further investigation. Current evidence indicates that anxiety-like behaviors are associated with excessive activation of mGluR5 and the downregulation of the BDNF/TrkB pathway. Our research demonstrates that S-ketamine effectively alleviates PTX-induced mechanical allodynia and anxiety-like behaviors. This therapeutic effect may be mediated through attenuating mGluR5-driven pyramidal overactivation and restoring the BDNF/TrkB signaling cascade, which collectively normalizes aberrant local neural oscillations. However, the precise causal sequence of this signaling cascade warrants further targeted investigation.

## Data Availability

The original contributions presented in the study are included in the article/Supplementary material, further inquiries can be directed to the corresponding author/s.
