# Supplementary material for: S-ketamine mitigates paclitaxel-induced pain-related anxiety-like behavior through downregulation of mGluR5 and activation of the BDNF/TrkB signaling pathway
Source: Front Neurol. 2026 Apr 23;17:1801549. doi: 10.3389/fneur.2026.1801549 (PMC13149193; doi:10.3389/fneur.2026.1801549)
Supplement: Supplementary file 1 [file Table_1.docx]

**Supplemental file1**

| **Animal** | **Source** | |
| --- | --- | --- |
| C57BL/6 mice | Changsheng Biotechnology Co. LTD, Liaoning, China | |
| **Product** | **Code** | **Manufacturer** |
| Sevoflurane | 23110331 | Shanghai Hengrui Pharmaceutical Co., Ltd, Shanghai, China |
| S-ketamine | 231021BL | Shanghai Hengrui Pharmaceutical Co., Ltd, Shanghai, China |
| Paclitaxel | P1632, | TCI, Tokyo, Japan |
| CHPG | HY-101304, | MedChemExpress, New Jersey, USA |
| paraformaldehyde | 20240306 | Beijing Yili Fine Chemicals Co., LTD, Beijing, China |
| Triton X-100 | T8200 | Solarbio, Beijing, China |
| Sodium citrate | P0083 | Beyotime, Shanghai, China |
| Quickblock^TM^ Blocking Buffer for Immunol Staining | P0260 | Beyotime, Shanghai, China |
| Mouse anti- c-Fos | ab208942 | Abcam |
| Rabbit anti- CaMKII | ab52476 | Abcam |
| Rabbit anti-mGluR5 | AF1744 | Beyotime, Shanghai, China |
| Rabbit anti-BDNF | AF1423 | Beyotime, Shanghai, China |
| Rabbit anti-PSD95 | AF1096 | Beyotime, Shanghai, China |
| Polyclonal rabbit anti-phosphorylated TrkB | AF1963 | Beyotime, Shanghai, China |
| Polyclonal rabbit anti-TrkB | GB11295-1-100 | Servicebio, Wuhan, China |
| CyTM3-Conjugated Goat Anti-Mouse IgG | A0521 | Beyotime, Shanghai, China |
| FITC goat anti-Rabbit IgG | A0562 | Beyotime, Shanghai, China |
| DAPI | P0131 | Beyotime, Shanghai, China |
| **Software** | **version** | **manufacturers** |
| Image-Pro | Plus 6.0 | NIH, Bethesda, MD, USA |
| Neuroexplorer | Version 5.0 | Plexon Inc., Dallas, TX |
| **Equipment** | **Version/Code** | **manufacturers** |
| Computerized tracking system Labmaze | Version 3.0 | Zhongshi Science & Technology, Beijing, China |
| Microfilament array electrodes | 2307051101MWA08–2 | Kedou Brain-Computer Technology Co., Ltd., Suzhou, China |
| Brain stereotaxic apparatus |  | Zhongshi Science & Technology, Beijing, China |
| NeuroLego amplifier |  | Jiangsu Brain Medical Technology Co. Ltd., Nanjing, China |
| Fluorescence microscope | CSIM110 | Sunny, Beijing, China |
