# Supplementary material for: S-ketamine mitigates paclitaxel-induced pain-related anxiety-like behavior through downregulation of mGluR5 and activation of the BDNF/TrkB signaling pathway
Source: Front Neurol. 2026 Apr 23;17:1801549. doi: 10.3389/fneur.2026.1801549 (PMC13149193; doi:10.3389/fneur.2026.1801549)
Supplement: Supplementary file 2 [file Table_2.docx]

**Fig.2**

| Mechanical withdrawal threshold (g) | | | | | | | | | Mechanical withdrawal  threshold (g) |
| --- | --- | --- | --- | --- | --- | --- | --- | --- | --- |
| Shapiro-Wilk test | Vehicle | | PTX | | S-katamine | | PTX+S-katamine | |  |
| W | 0.9005 | | 0.8577 | | 0.9492 | | 0.9375 | |  |
| P value | 0.3343 | | 0.1442 | | 0.7229 | | 0.6160 | |  |
| Passed normality test (alpha=0.05)? | Yes | | Yes | | Yes | | Yes | |  |
| P value summary | ns | | ns | | ns | | ns | |  |
|  | | | | | | | | |  |
| ANOVA table | SS | DF | | MS | | F (DFn, DFd) | | P value |  |
| Interaction | 18.57 | 18 | | 1.031 | | F (18, 308) = 62.31 | | P<0.0001 |  |
| Row factor | 9.587 | 6 | | 1.598 | | F (6, 308) = 96.53 | | P<0.0001 |  |
| Column factor | 49.75 | 3 | | 16.58 | | F (3, 308) = 1002 | | P<0.0001 |  |
| Residual | 5.099 | 308 | | 0.01655 | |  | |  |  |

| Total distance (cm) | | | | | | | | |
| --- | --- | --- | --- | --- | --- | --- | --- | --- |
| Shapiro-Wilk test | Vehicle | | PTX | | S-katamine | | PTX+S-katamine | |
| W | 0.9516 | | 0.9634 | | 0.9177 | | 0.9415 | |
| P value | 0.6601 | | 0.8314 | | 0.2672 | | 0.5174 | |
| Passed normality test (alpha=0.05)? | Yes | | Yes | | Yes | | Yes | |
| P value summary | ns | | ns | | ns | | ns | |
|  | | | | | | | | |
| ANOVA table | SS | DF | | MS | | F (DFn, DFd) | | P value |
| Treatment (between columns) | 411833 | 3 | | 137278 | | F (3, 44) = 1.532 | | P=0.2196 |
| Residual (within columns) | 3943169 | 44 | | 89617 | |  | |  |
|  | | | | | | | | |
| Tukey's multiple comparisons test | Mean Diff. | 95.00% CI of diff. | | Below threshold? | | Summary | | Adjusted P Value |
| Vehicle vs. PTX | 225.3 | -101.1 to 551.6 | | No | | ns | | 0.2674 |
| Vehicle vs. S-katamine | -0.4008 | -326.7 to 325.9 | | No | | ns | | >0.9999 |
| Vehicle vs. PTX+S-katamine | 98.99 | -227.3 to 425.3 | | No | | ns | | 0.8494 |
| PTX vs. S-katamine | -225.7 | -552.0 to 100.7 | | No | | ns | | 0.2660 |
| PTX vs. PTX+S-katamine | -126.3 | -452.6 to 200.1 | | No | | ns | | 0.7311 |
| S-katamine vs. PTX+S-katamine | 99.39 | -226.9 to 425.7 | | No | | ns | | 0.8479 |

| Distance in central area（% of total distance) | | | | | | | | |
| --- | --- | --- | --- | --- | --- | --- | --- | --- |
| Shapiro-Wilk test | Vehicle | | PTX | | S-katamine | | PTX+S-katamine | |
| W | 0.9545 | | 0.9807 | | 0.9649 | | 0.9582 | |
| P value | 0.7036 | | 0.9863 | | 0.8506 | | 0.7574 | |
| Passed normality test (alpha=0.05)? | Yes | | Yes | | Yes | | Yes | |
| P value summary | ns | | ns | | ns | | ns | |
|  | | | | | | | | |
| ANOVA table | SS | DF | | MS | | F (DFn, DFd) | | P value |
| Treatment (between columns) | 359.0 | 3 | | 119.7 | | F (3, 44) = 21.74 | | P<0.0001 |
| Residual (within columns) | 242.2 | 44 | | 5.504 | |  | |  |
|  | | | | | | | | |
| Tukey's multiple comparisons test | Mean Diff. | 95.00% CI of diff. | | Below threshold? | | Summary | | Adjusted P Value |
| Vehicle vs. PTX | 6.990 | 4.433 to 9.547 | | Yes | | **** | | <0.0001 |
| Vehicle vs. S-katamine | 0.7608 | -1.796 to 3.318 | | No | | ns | | 0.8566 |
| Vehicle vs. PTX+S-katamine | 3.401 | 0.8437 to 5.958 | | Yes | | ** | | 0.0050 |
| PTX vs. S-katamine | -6.229 | -8.786 to -3.672 | | Yes | | **** | | <0.0001 |
| PTX vs. PTX+S-katamine | -3.589 | -6.146 to -1.032 | | Yes | | ** | | 0.0028 |
| S-katamine vs. PTX+S-katamine | 2.640 | 0.08285 to 5.197 | | Yes | | * | | 0.0407 |

| Entries in open arm （% of total entries） | | | | | | | | |
| --- | --- | --- | --- | --- | --- | --- | --- | --- |
| Shapiro-Wilk test | Vehicle | | PTX | | S-katamine | | PTX+S-katamine | |
| W | 0.9741 | | 0.9598 | | 0.9193 | | 0.9280 | |
| P value | 0.9486 | | 0.7808 | | 0.2798 | | 0.3589 | |
| Passed normality test (alpha=0.05)? | Yes | | Yes | | Yes | | Yes | |
| P value summary | ns | | ns | | ns | | ns | |
|  | | | | | | | | |
| ANOVA table | SS | DF | | MS | | F (DFn, DFd) | | P value |
| Treatment (between columns) | 2630 | 3 | | 876.5 | | F (3, 44) = 14.32 | | P<0.0001 |
| Residual (within columns) | 2694 | 44 | | 61.23 | |  | |  |
|  | | | | | | | | |
| Tukey's multiple comparisons test | Mean Diff. | 95.00% CI of diff. | | Below threshold? | | Summary | | Adjusted P Value |
| Vehicle vs. PTX | 20.01 | 11.48 to 28.54 | | Yes | | **** | | <0.0001 |
| Vehicle vs. S-katamine | 4.998 | -3.531 to 13.53 | | No | | ns | | 0.4089 |
| Vehicle vs. PTX+S-katamine | 10.03 | 1.497 to 18.55 | | Yes | | * | | 0.0155 |
| PTX vs. S-katamine | -15.02 | -23.54 to -6.486 | | Yes | | *** | | 0.0001 |
| PTX vs. PTX+S-katamine | -9.988 | -18.52 to -1.458 | | Yes | | * | | 0.0160 |
| S-katamine vs. PTX+S-katamine | 5.027 | -3.502 to 13.56 | | No | | ns | | 0.4037 |

| Time in open arm （% of total time） | | | | | | | | |
| --- | --- | --- | --- | --- | --- | --- | --- | --- |
| Shapiro-Wilk test | Vehicle | | PTX | | S-katamine | | PTX+S-katamine | |
| W | 0.9408 | | 0.9293 | | 0.9395 | | 0.9511 | |
| P value | 0.5079 | | 0.3727 | | 0.4922 | | 0.6531 | |
| Passed normality test (alpha=0.05)? | Yes | | Yes | | Yes | | Yes | |
| P value summary | ns | | ns | | ns | | ns | |
|  | | | | | | | | |
| ANOVA table | SS | DF | | MS | | F (DFn, DFd) | | P value |
| Treatment (between columns) | 5370 | 3 | | 1790 | | F (3, 44) = 46.24 | | P<0.0001 |
| Residual (within columns) | 1703 | 44 | | 38.71 | |  | |  |
|  | | | | | | | | |
| Tukey's multiple comparisons test | Mean Diff. | 95.00% CI of diff. | | Below threshold? | | Summary | | Adjusted P Value |
| Vehicle vs. PTX | 27.72 | 20.94 to 34.50 | | Yes | | **** | | <0.0001 |
| Vehicle vs. S-katamine | 7.537 | 0.7561 to 14.32 | | Yes | | * | | 0.0241 |
|  | 18.74 | 11.96 to 25.52 | | Yes | | **** | | <0.0001 |
| PTX vs. S-katamine | -20.18 | -26.96 to -13.40 | | Yes | | **** | | <0.0001 |
| PTX vs. PTX+S-katamine | -8.979 | -15.76 to -2.198 | | Yes | | ** | | 0.0052 |
| S-katamine vs. PTX+S-katamine | 11.20 | 4.422 to 17.98 | | Yes | | *** | | 0.0004 |

**Fig.3**

| Faring rate (Hz) | | | | | | | | |
| --- | --- | --- | --- | --- | --- | --- | --- | --- |
| Shapiro-Wilk test | Vehicle | | PTX | | S-katamine | | PTX+S-katamine | |
| W | 0.9241 | | 0.9456 | | 0.9756 | | 0.9926 | |
| P value | 0.4670 | | 0.5505 | | 0.7005 | | 0.8354 | |
| Passed normality test (alpha=0.05)? | Yes | | Yes | | Yes | | Yes | |
| P value summary | ns | | ns | | ns | | ns | |
|  | | | | | | | | |
| ANOVA table | SS | DF | | MS | | F (DFn, DFd) | | P value |
| Treatment (between columns) | 21.18 | 3 | | 7.059 | | F (3, 8) = 14.28 | | P=0.0014 |
| Residual (within columns) | 3.955 | 8 | | 0.4944 | |  | |  |
|  | | | | | | | | |
| Tukey's multiple comparisons test | Mean Diff. | 95.00% CI of diff. | | Below threshold? | | Summary | | Adjusted P Value |
| Vehicle vs. PTX | -3.343 | -5.182 to -1.505 | | Yes | | ** | | 0.0018 |
| Vehicle vs. S-katamine | -0.1867 | -2.025 to 1.652 | | No | | ns | | 0.9872 |
| Vehicle vs. PTX+S-katamine | -1.157 | -2.995 to 0.6819 | | No | | ns | | 0.2591 |
| PTX vs. S-katamine | 3.157 | 1.318 to 4.995 | | Yes | | ** | | 0.0026 |
| PTX vs. PTX+S-katamine | 2.187 | 0.3481 to 4.025 | | Yes | | * | | 0.0216 |
| S-katamine vs. PTX+S-katamine | -0.9700 | -2.809 to 0.8685 | | No | | ns | | 0.3878 |

| Normallization of PSD (dB) | | | | | | | | |
| --- | --- | --- | --- | --- | --- | --- | --- | --- |
| Shapiro-Wilk test | Vehicle | | PTX | | S-katamine | | PTX+S-katamine | |
| W | 0.9187 | | 0.8710 | | 0.9404 | | 0.9721 | |
| P value | 0.4479 | | 0.2983 | | 0.5288 | | 0.6795 | |
| Passed normality test (alpha=0.05)? | Yes | | Yes | | Yes | | Yes | |
| P value summary | ns | | ns | | ns | | ns | |
|  | | | | | | | | |
| ANOVA table | SS | DF | | MS | | F (DFn, DFd) | | P value |
| Treatment (between columns) | 256.3 | 3 | | 85.42 | | F (3, 8) = 63.45 | | P<0.0001 |
| Residual (within columns) | 10.77 | 8 | | 1.346 | |  | |  |
|  | | | | | | | | |
| Tukey's multiple comparisons test | Mean Diff. | 95.00% CI of diff. | | Below threshold? | | Summary | | Adjusted P Value |
| Vehicle vs. PTX | -11.00 | -14.04 to -7.969 | | Yes | | **** | | <0.0001 |
| Vehicle vs. S-katamine | 0.5200 | -2.514 to 3.554 | | No | | ns | | 0.9443 |
| Vehicle vs. PTX+S-katamine | -4.470 | -7.504 to -1.436 | | Yes | | ** | | 0.0066 |
| PTX vs. S-katamine | 11.52 | 8.489 to 14.56 | | Yes | | **** | | <0.0001 |
| PTX vs. PTX+S-katamine | 6.533 | 3.499 to 9.567 | | Yes | | *** | | 0.0006 |
| S-katamine vs. PTX+S-katamine | -4.990 | -8.024 to -1.956 | | Yes | | ** | | 0.0034 |

| Normallization of PSD (dB) | | | | | | | | |
| --- | --- | --- | --- | --- | --- | --- | --- | --- |
| Shapiro-Wilk test | Vehicle | | PTX | | S-katamine | | PTX+S-katamine | |
| W | 0.9185 | | 0.9979 | | 0.9304 | | 0.9792 | |
| P value | 0.4470 | | 0.9127 | | 0.4902 | | 0.7233 | |
| Passed normality test (alpha=0.05)? | Yes | | Yes | | Yes | | Yes | |
| P value summary | ns | | ns | | ns | | ns | |
|  | | | | | | | | |
| ANOVA table | SS | DF | | MS | | F (DFn, DFd) | | P value |
| Treatment (between columns) | 802.9 | 3 | | 267.6 | | F (3, 8) = 128.5 | | P<0.0001 |
| Residual (within columns) | 16.66 | 8 | | 2.083 | |  | |  |
|  | | | | | | | | |
| Tukey's multiple comparisons test | Mean Diff. | 95.00% CI of diff. | | Below threshold? | | Summary | | Adjusted P Value |
| Vehicle vs. PTX | -19.57 | -23.35 to -15.80 | | Yes | | **** | | <0.0001 |
| Vehicle vs. S-katamine | 0.8933 | -2.880 to 4.667 | | No | | ns | | 0.8708 |
| Vehicle vs. PTX+S-katamine | -6.423 | -10.20 to -2.650 | | Yes | | ** | | 0.0027 |
| PTX vs. S-katamine | 20.47 | 16.69 to 24.24 | | Yes | | **** | | <0.0001 |
| PTX vs. PTX+S-katamine | 13.15 | 9.377 to 16.92 | | Yes | | **** | | <0.0001 |
| S-katamine vs. PTX+S-katamine | -7.317 | -11.09 to -3.543 | | Yes | | ** | | 0.0012 |

| Maximal modulation index (×10^-4^） | | | | | | | | |
| --- | --- | --- | --- | --- | --- | --- | --- | --- |
| Shapiro-Wilk test | Vehicle | | PTX | | S-katamine | | PTX+S-katamine | |
| W | 0.8041 | | 0.9464 | | 0.8536 | | 0.8982 | |
| P value | 0.1244 | | 0.5539 | | 0.2502 | | 0.3797 | |
| Passed normality test (alpha=0.05)? | Yes | | Yes | | Yes | | Yes | |
| P value summary | ns | | ns | | ns | | ns | |
|  | | | | | | | | |
| ANOVA table | SS | DF | | MS | | F (DFn, DFd) | | P value |
| Treatment (between columns) | 792.9 | 3 | | 264.3 | | F (3, 8) = 12.45 | | P=0.0022 |
| Residual (within columns) | 169.8 | 8 | | 21.23 | |  | |  |
|  | | | | | | | | |
| Tukey's multiple comparisons test | Mean Diff. | 95.00% CI of diff. | | Below threshold? | | Summary | | Adjusted P Value |
| Vehicle vs. PTX | 20.58 | 8.536 to 32.63 | | Yes | | ** | | 0.0026 |
| Vehicle vs. S-katamine | 1.440 | -10.61 to 13.49 | | No | | ns | | 0.9796 |
| Vehicle vs. PTX+S-katamine | 7.897 | -4.151 to 19.94 | | No | | ns | | 0.2320 |
| PTX vs. S-katamine | -19.14 | -31.19 to -7.096 | | Yes | | ** | | 0.0042 |
| PTX vs. PTX+S-katamine | -12.69 | -24.73 to -0.6390 | | Yes | | * | | 0.0394 |
| S-katamine vs. PTX+S-katamine | 6.457 | -5.591 to 18.50 | | No | | ns | | 0.3758 |

**Fig.4**

| c-Fos positive CaMK II（%） | | | | | | | | |
| --- | --- | --- | --- | --- | --- | --- | --- | --- |
| Shapiro-Wilk test | Vehicle | | PTX | | S-katamine | | PTX+S-katamine | |
| W | 0.9714 | | 0.9436 | | 0.9517 | | 0.9891 | |
| P value | 0.9018 | | 0.6880 | | 0.7537 | | 0.9868 | |
| Passed normality test (alpha=0.05)? | Yes | | Yes | | Yes | | Yes | |
| P value summary | ns | | ns | | ns | | ns | |
|  | | | | | | | | |
| ANOVA table | SS | DF | | MS | | F (DFn, DFd) | | P value |
| Treatment (between columns) | 6483 | 3 | | 2161 | | F (3, 20) = 47.93 | | P<0.0001 |
| Residual (within columns) | 901.7 | 20 | | 45.08 | |  | |  |
|  | | | | | | | | |
| Tukey's multiple comparisons test | Mean Diff. | 95.00% CI of diff. | | Below threshold? | | Summary | | Adjusted P Value |
| Vehicle vs. PTX | -19.58 | -28.09 to -11.07 | | Yes | | **** | | <0.0001 |
| Vehicle vs. S-katamine | 0.8217 | -7.688 to 9.332 | | No | | ns | | 0.9929 |
| Vehicle vs. PTX+S-katamine | -7.655 | -16.16 to 0.8548 | | No | | ns | | 0.0875 |
| PTX vs. S-katamine | 20.40 | 11.89 to 28.91 | | Yes | | **** | | <0.0001 |
| PTX vs. PTX+S-katamine | 11.93 | 3.417 to 20.44 | | Yes | | ** | | 0.0043 |
| S-katamine vs. PTX+S-katamine | -8.477 | -16.99 to 0.03318 | | No | | ns | | 0.0511 |

| mGluR5 intensity (% Vehicle) | | | | | | | | |
| --- | --- | --- | --- | --- | --- | --- | --- | --- |
| Shapiro-Wilk test | Vehicle | | PTX | | S-katamine | | PTX+S-katamine | |
| W | 0.9664 | | 0.9325 | | 0.9348 | | 0.9171 | |
| P value | 0.8673 | | 0.5993 | | 0.6178 | | 0.4844 | |
| Passed normality test (alpha=0.05)? | Yes | | Yes | | Yes | | Yes | |
| P value summary | ns | | ns | | ns | | ns | |
|  | | | | | | | | |
| ANOVA table | SS | DF | | MS | | F (DFn, DFd) | | P value |
| Treatment (between columns) | 2.222 | 3 | | 0.7406 | | F (3, 20) = 35.48 | | P<0.0001 |
| Residual (within columns) | 0.4174 | 20 | | 0.02087 | |  | |  |
|  | | | | | | | | |
| Tukey's multiple comparisons test | Mean Diff. | 95.00% CI of diff. | | Below threshold? | | Summary | | Adjusted P Value |
| Vehicle vs. PTX | -0.7050 | -0.9385 to -0.4715 | | Yes | | **** | | <0.0001 |
| Vehicle vs. S-katamine | 0.001667 | -0.2318 to 0.2351 | | No | | ns | | >0.9999 |
| Vehicle vs. PTX+S-katamine | -0.4600 | -0.6935 to -0.2265 | | Yes | | *** | | 0.0001 |
| PTX vs. S-katamine | 0.7067 | 0.4732 to 0.9401 | | Yes | | **** | | <0.0001 |
| PTX vs. PTX+S-katamine | 0.2450 | 0.01154 to 0.4785 | | Yes | | * | | 0.0376 |
| S-katamine vs. PTX+S-katamine | -0.4617 | -0.6951 to -0.2282 | | Yes | | *** | | 0.0001 |

| BDNF intensity in the mPFC (% Vehicle) | | | | | | | | |
| --- | --- | --- | --- | --- | --- | --- | --- | --- |
| Shapiro-Wilk test | Vehicle | | PTX | | S-katamine | | PTX+S-katamine | |
| W | 0.9429 | | 0.8792 | | 0.9710 | | 0.9899 | |
| P value | 0.6827 | | 0.2655 | | 0.8988 | | 0.9889 | |
| Passed normality test (alpha=0.05)? | Yes | | Yes | | Yes | | Yes | |
| P value summary | ns | | ns | | ns | | ns | |
|  | | | | | | | | |
| ANOVA table | SS | DF | | MS | | F (DFn, DFd) | | P value |
| Treatment (between columns) | 10446 | 3 | | 3482 | | F (3, 20) = 46.52 | | P<0.0001 |
| Residual (within columns) | 1497 | 20 | | 74.85 | |  | |  |
|  | | | | | | | | |
| Tukey's multiple comparisons test | Mean Diff. | 95.00% CI of diff. | | Below threshold? | | Summary | | Adjusted P Value |
| Vehicle vs. PTX | 47.15 | 33.17 to 61.13 | | Yes | | **** | | <0.0001 |
| Vehicle vs. S-katamine | -5.843 | -19.82 to 8.137 | | No | | ns | | 0.6520 |
| Vehicle vs. PTX+S-katamine | 22.14 | 8.161 to 36.12 | | Yes | | ** | | 0.0013 |
| PTX vs. S-katamine | -52.99 | -66.97 to -39.01 | | Yes | | **** | | <0.0001 |
| PTX vs. PTX+S-katamine | -25.01 | -38.99 to -11.03 | | Yes | | *** | | 0.0004 |
| S-katamine vs. PTX+S-katamine | 27.99 | 14.00 to 41.97 | | Yes | | **** | | <0.0001 |

| PSD95 intensity in the mPFC (% Vehicle) | | | | | | | | |
| --- | --- | --- | --- | --- | --- | --- | --- | --- |
| Shapiro-Wilk test | Vehicle | | PTX | | S-katamine | | PTX+S-katamine | |
| W | 0.9175 | | 0.9189 | | 0.9290 | | 0.9652 | |
| P value | 0.4873 | | 0.4977 | | 0.5727 | | 0.8590 | |
| Passed normality test (alpha=0.05)? | Yes | | Yes | | Yes | | Yes | |
| P value summary | ns | | ns | | ns | | ns | |
|  | | | | | | | | |
| ANOVA table | SS | DF | | MS | | F (DFn, DFd) | | P value |
| Treatment (between columns) | 12832 | 3 | | 4277 | | F (3, 20) = 37.95 | | P<0.0001 |
| Residual (within columns) | 2254 | 20 | | 112.7 | |  | |  |
|  | | | | | | | | |
| Tukey's multiple comparisons test | Mean Diff. | 95.00% CI of diff. | | Below threshold? | | Summary | | Adjusted P Value |
| Vehicle vs. PTX | 56.15 | 38.99 to 73.30 | | Yes | | **** | | <0.0001 |
| Vehicle vs. S-katamine | -0.8483 | -18.00 to 16.31 | | No | | ns | | 0.9990 |
| Vehicle vs. PTX+S-katamine | 20.98 | 3.820 to 38.13 | | Yes | | * | | 0.0132 |
| PTX vs. S-katamine | -56.99 | -74.15 to -39.84 | | Yes | | **** | | <0.0001 |
| PTX vs. PTX+S-katamine | -35.17 | -52.33 to -18.01 | | Yes | | **** | | <0.0001 |
| S-katamine vs. PTX+S-katamine | 21.82 | 4.668 to 38.98 | | Yes | | ** | | 0.0097 |

| Ratio of mGluR5/GAPDH | | | | | | | | |
| --- | --- | --- | --- | --- | --- | --- | --- | --- |
| Shapiro-Wilk test | Vehicle | | PTX | | S-katamine | | PTX+S-katamine | |
| W | 0.9608 | | 0.9463 | | 0.9635 | | 0.9834 | |
| P value | 0.8261 | | 0.7100 | | 0.8465 | | 0.9670 | |
| Passed normality test (alpha=0.05)? | Yes | | Yes | | Yes | | Yes | |
| P value summary | ns | | ns | | ns | | ns | |
|  | | | | | | | | |
| ANOVA table | SS | DF | | MS | | F (DFn, DFd) | | P value |
| Treatment (between columns) | 0.7382 | 3 | | 0.2461 | | F (3, 20) = 35.25 | | P<0.0001 |
| Residual (within columns) | 0.1396 | 20 | | 0.006980 | |  | |  |
|  | | | | | | | | |
| Tukey's multiple comparisons test | Mean Diff. | 95.00% CI of diff. | | Below threshold? | | Summary | | Adjusted P Value |
| Vehicle vs. PTX | -0.4267 | -0.5617 to -0.2917 | | Yes | | **** | | <0.0001 |
| Vehicle vs. S-katamine | -0.03667 | -0.1717 to 0.09834 | | No | | ns | | 0.8713 |
| Vehicle vs. PTX+S-katamine | -0.2767 | -0.4117 to -0.1417 | | Yes | | **** | | <0.0001 |
| PTX vs. S-katamine | 0.3900 | 0.2550 to 0.5250 | | Yes | | **** | | <0.0001 |
| PTX vs. PTX+S-katamine | 0.1500 | 0.01499 to 0.2850 | | Yes | | * | | 0.0261 |
| S-katamine vs. PTX+S-katamine | -0.2400 | -0.3750 to -0.1050 | | Yes | | *** | | 0.0004 |

| Ratio of BDNF/GAPDH | | | | | | | | |
| --- | --- | --- | --- | --- | --- | --- | --- | --- |
| Shapiro-Wilk test | Vehicle | | PTX | | S-katamine | | PTX+S-katamine | |
| W | 0.9334 | | 0.9024 | | 0.9548 | | 0.8698 | |
| P value | 0.6062 | | 0.3882 | | 0.7786 | | 0.2255 | |
| Passed normality test (alpha=0.05)? | Yes | | Yes | | Yes | | Yes | |
| P value summary | ns | | ns | | ns | | ns | |
|  | | | | | | | | |
| ANOVA table | SS | DF | | MS | | F (DFn, DFd) | | P value |
| Treatment (between columns) | 0.3853 | 3 | | 0.1284 | | F (3, 20) = 13.64 | | P<0.0001 |
| Residual (within columns) | 0.1884 | 20 | | 0.009419 | |  | |  |
|  | | | | | | | | |
| Tukey's multiple comparisons test | Mean Diff. | 95.00% CI of diff. | | Below threshold? | | Summary | | Adjusted P Value |
| Vehicle vs. PTX | 0.2550 | 0.09817 to 0.4118 | | Yes | | ** | | 0.0010 |
| Vehicle vs. S-katamine | -0.09000 | -0.2468 to 0.06683 | | No | | ns | | 0.3978 |
| Vehicle vs. PTX+S-katamine | 0.04000 | -0.1168 to 0.1968 | | No | | ns | | 0.8904 |
| PTX vs. S-katamine | -0.3450 | -0.5018 to -0.1882 | | Yes | | **** | | <0.0001 |
| PTX vs. PTX+S-katamine | -0.2150 | -0.3718 to -0.05817 | | Yes | | ** | | 0.0052 |
| S-katamine vs. PTX+S-katamine | 0.1300 | -0.02683 to 0.2868 | | No | | ns | | 0.1269 |

| p-TrkB/TrkB | | | | | | | | |
| --- | --- | --- | --- | --- | --- | --- | --- | --- |
| Shapiro-Wilk test | Vehicle | | PTX | | S-katamine | | PTX+S-katamine | |
| W | 0.8597 | | 0.9369 | | 0.8793 | | 0.8848 | |
| P value | 0.1881 | | 0.6346 | | 0.2659 | | 0.2920 | |
| Passed normality test (alpha=0.05)? | Yes | | Yes | | Yes | | Yes | |
| P value summary | ns | | ns | | ns | | ns | |
|  | | | | | | | | |
| ANOVA table | SS | DF | | MS | | F (DFn, DFd) | | P value |
| Treatment (between columns) | 0.2648 | 3 | | 0.08828 | | F (3, 20) = 18.60 | | P<0.0001 |
| Residual (within columns) | 0.09492 | 20 | | 0.004746 | |  | |  |
|  | | | | | | | | |
| Tukey's multiple comparisons test | Mean Diff. | 95.00% CI of diff. | | Below threshold? | | Summary | | Adjusted P Value |
| Vehicle vs. PTX | 0.2733 | 0.1620 to 0.3847 | | Yes | | **** | | <0.0001 |
| Vehicle vs. S-katamine | 0.04000 | -0.07132 to 0.1513 | | No | | ns | | 0.7479 |
| Vehicle vs. PTX+S-katamine | 0.1317 | 0.02034 to 0.2430 | | Yes | | * | | 0.0169 |
| PTX vs. S-katamine | -0.2333 | -0.3447 to -0.1220 | | Yes | | **** | | <0.0001 |
| PTX vs. PTX+S-katamine | -0.1417 | -0.2530 to -0.03034 | | Yes | | ** | | 0.0097 |
| S-katamine vs. PTX+S-katamine | 0.09167 | -0.01966 to 0.2030 | | No | | ns | | 0.1305 |

| Ratio of PSD95/GAPDH | | | | | | | | |
| --- | --- | --- | --- | --- | --- | --- | --- | --- |
| Shapiro-Wilk test | Vehicle | | PTX | | S-katamine | | PTX+S-katamine | |
| W | 0.9044 | | 0.9277 | | 0.8039 | | 0.9565 | |
| P value | 0.4005 | | 0.5626 | | 0.0637 | | 0.7921 | |
| Passed normality test (alpha=0.05)? | Yes | | Yes | | Yes | | Yes | |
| P value summary | ns | | ns | | ns | | ns | |
|  | | | | | | | | |
| ANOVA table | SS | DF | | MS | | F (DFn, DFd) | | P value |
| Treatment (between columns) | 0.4414 | 3 | | 0.1471 | | F (3, 20) = 13.30 | | P<0.0001 |
| Residual (within columns) | 0.2213 | 20 | | 0.01107 | |  | |  |
|  | | | | | | | | |
| Tukey's multiple comparisons test | Mean Diff. | 95.00% CI of diff. | | Below threshold? | | Summary | | Adjusted P Value |
| Vehicle vs. PTX | 0.3317 | 0.1617 to 0.5017 | | Yes | | *** | | 0.0001 |
| Vehicle vs. S-katamine | 0.006667 | -0.1633 to 0.1767 | | No | | ns | | 0.9995 |
| Vehicle vs. PTX+S-katamine | 0.1600 | -0.009990 to 0.3300 | | No | | ns | | 0.0696 |
| PTX vs. S-katamine | -0.3250 | -0.4950 to -0.1550 | | Yes | | *** | | 0.0002 |
| PTX vs. PTX+S-katamine | -0.1717 | -0.3417 to -0.001676 | | Yes | | * | | 0.0473 |
| S-katamine vs. PTX+S-katamine | 0.1533 | -0.01666 to 0.3233 | | No | | ns | | 0.0863 |

**Fig.5**

| Total distance (cm) | | | |
| --- | --- | --- | --- |
| Shapiro-Wilk test | PTX+S +V | PTX+S +C | |
| W | 0.9620 | 0.8954 | |
| P value | 0.8115 | 0.1383 | |
| Passed normality test (alpha=0.05)? | Yes | Yes | |
| P value summary | ns | ns | |
|  | | | |
| Unpaired t test | |  |  |
| P value | | 0.4442 |  |
| P value summary | | ns |  |
| Significantly different (P < 0.05)? | | No |  |
| One- or two-tailed P value? | | Two-tailed |  |
| t, df | | t=0.7791, df=22 |  |

| Distance in central area（% of total distance) | | | |
| --- | --- | --- | --- |
| Shapiro-Wilk test | PTX+S +V | PTX+S +C | |
| W | 0.9571 | 0.9641 | |
| P value | 0.7422 | 0.8408 | |
| Passed normality test (alpha=0.05)? | Yes | Yes | |
| P value summary | ns | ns | |
|  | | | |
| Unpaired t test | |  |  |
| P value | | 10.34 |  |
| P value summary | | 5.549 |  |
| Significantly different (P < 0.05)? | | -4.792 ± 0.7828 |  |
| One- or two-tailed P value? | | -6.415 to -3.168 |  |
| t, df | | 0.6301 |  |

| Entries in open arm （% of total entries） | | | |
| --- | --- | --- | --- |
| Shapiro-Wilk test | PTX+S +V | PTX+S +C | |
| W | 0.9674 | 0.9704 | |
| P value | 0.8813 | 0.9154 | |
| Passed normality test (alpha=0.05)? | Yes | Yes | |
| P value summary | ns | ns | |
|  | | | |
| Unpaired t test | |  |  |
| P value | | <0.0001 |  |
| P value summary | | **** |  |
| Significantly different (P < 0.05)? | | Yes |  |
| One- or two-tailed P value? | | Two-tailed |  |
| t, df | | t=5.400, df=22 |  |

| Time in open arm （% of total time） | | | |
| --- | --- | --- | --- |
| Shapiro-Wilk test | PTX+S +V | PTX+S +C | |
| W | 0.9841 | 0.9558 | |
| P value | 0.9950 | 0.7226 | |
| Passed normality test (alpha=0.05)? | Yes | Yes | |
| P value summary | ns | ns | |
|  | | | |
| Unpaired t test | |  |  |
| P value | | <0.0001 |  |
| P value summary | | **** |  |
| Significantly different (P < 0.05)? | | Yes |  |
| One- or two-tailed P value? | | Two-tailed |  |
| t, df | | t=6.162, df=22 |  |

| Faring rate (Hz) | | | |
| --- | --- | --- | --- |
| Shapiro-Wilk test | PTX+S +V | PTX+S +C | |
| W | 0.9356 | 0.9501 | |
| P value | 0.5098 | 0.5696 | |
| Passed normality test (alpha=0.05)? | Yes | Yes | |
| P value summary | ns | ns | |
|  | | | |
| Unpaired t test | |  |  |
| P value | | 0.0029 |  |
| P value summary | | ** |  |
| Significantly different (P < 0.05)? | | Yes |  |
| One- or two-tailed P value? | | Two-tailed |  |
| t, df | | t=6.518, df=4 |  |

| Normallization of PSD (dB) | | | |
| --- | --- | --- | --- |
| Shapiro-Wilk test | PTX+S +V | PTX+S +C | |
| W | 0.8856 | 0.9471 | |
| P value | 0.3409 | 0.5569 | |
| Passed normality test (alpha=0.05)? | Yes | Yes | |
| P value summary | ns | ns | |
|  | | | |
| Unpaired t test | |  |  |
| P value | | 0.0012 |  |
| P value summary | | ** |  |
| Significantly different (P < 0.05)? | | Yes |  |
| One- or two-tailed P value? | | Two-tailed |  |
| t, df | | t=8.234, df=4 |  |

| Normallization of PSD (dB) | | | |
| --- | --- | --- | --- |
| Shapiro-Wilk test | PTX+S +V | PTX+S +C | |
| W | 0.9779 | 0.9843 | |
| P value | 0.7151 | 0.7601 | |
| Passed normality test (alpha=0.05)? | Yes | Yes | |
| P value summary | ns | ns | |
|  | | | |
| Unpaired t test | |  |  |
| P value | | <0.0001 |  |
| P value summary | | **** |  |
| Significantly different (P < 0.05)? | | Yes |  |
| One- or two-tailed P value? | | Two-tailed |  |
| t, df | | t=21.11, df=4 |  |

| Maximal modulation index (×10-4） | | | |
| --- | --- | --- | --- |
| Shapiro-Wilk test | PTX+S +V | PTX+S +C | |
| W | 0.9267 | 0.8883 | |
| P value | 0.4765 | 0.3492 | |
| Passed normality test (alpha=0.05)? | Yes | Yes | |
| P value summary | ns | ns | |
|  | | | |
| Unpaired t test | |  |  |
| P value | | 0.0055 |  |
| P value summary | | ** |  |
| Significantly different (P < 0.05)? | | Yes |  |
| One- or two-tailed P value? | | Two-tailed |  |
| t, df | | t=5.440, df=4 |  |

**Fig.6**

| c-Fos positive CaMKII（%） | | | |
| --- | --- | --- | --- |
| Shapiro-Wilk test | PTX+S +V | PTX+S +C | |
| W | 0.8683 | 0.8195 | |
| P value | 0.2194 | 0.0873 | |
| Passed normality test (alpha=0.05)? | Yes | Yes | |
| P value summary | ns | ns | |
|  | | | |
| Unpaired t test | |  |  |
| P value | | <0.0001 |  |
| P value summary | | **** |  |
| Significantly different (P < 0.05)? | | Yes |  |
| One- or two-tailed P value? | | Two-tailed |  |
| t, df | | t=7.202, df=10 |  |

| mGluR5 intensity (% PTX + S + V) | | | |
| --- | --- | --- | --- |
| Shapiro-Wilk test | PTX+S +V | PTX+S +C | |
| W | 0.9809 | 0.9594 | |
| P value | 0.9558 | 0.8152 | |
| Passed normality test (alpha=0.05)? | Yes | Yes | |
| P value summary | ns | ns | |
|  | | | |
| Unpaired t test | |  |  |
| P value | | 0.0125 |  |
| P value summary | | * |  |
| Significantly different (P < 0.05)? | | Yes |  |
| One- or two-tailed P value? | | Two-tailed |  |
| t, df | | t=3.038, df=10 |  |
| BDNF intensity in the mPFC (% PTX + S +V) | | | |
| Shapiro-Wilk test | PTX+S +V | PTX+S +C | |
| W | 0.9810 | 0.9721 | |
| P value | 0.9565 | 0.9061 | |
| Passed normality test (alpha=0.05)? | Yes | Yes | |
| P value summary | ns | ns | |
|  | | | |
| Unpaired t test | |  |  |
| P value | | <0.0001 |  |
| P value summary | | **** |  |
| Significantly different (P < 0.05)? | | Yes |  |
| One- or two-tailed P value? | | Two-tailed |  |
| t, df | | t=9.273, df=10 |  |

| PSD95 intensity in the mPFC (% PTX + S +V)） | | | |
| --- | --- | --- | --- |
| Shapiro-Wilk test | PTX+S +V | PTX+S +C | |
| W | 0.9246 | 0.9755 | |
| P value | 0.5391 | 0.9271 | |
| Passed normality test (alpha=0.05)? | Yes | Yes | |
| P value summary | ns | ns | |
|  | | | |
| Unpaired t test | |  |  |
| P value | | 0.0033 |  |
| P value summary | | ** |  |
| Significantly different (P < 0.05)? | | Yes |  |
| One- or two-tailed P value? | | Two-tailed |  |
| t, df | | t=3.827, df=10 |  |

| Ratio of mGluR5/GAPDH | | | |
| --- | --- | --- | --- |
| Shapiro-Wilk test | PTX+S +V | PTX+S +C | |
| W | 0.9379 | 0.9089 | |
| P value | 0.6425 | 0.4289 | |
| Passed normality test (alpha=0.05)? | Yes | Yes | |
| P value summary | ns | ns | |
|  | | | |
| Unpaired t test | |  |  |
| P value | | 0.0048 |  |
| P value summary | | ** |  |
| Significantly different (P < 0.05)? | | Yes |  |
| One- or two-tailed P value? | | Two-tailed |  |
| t, df | | t=3.602, df=10 |  |

| Ratio of BDNF/GAPDH） | | | |
| --- | --- | --- | --- |
| Shapiro-Wilk test | PTX+S +V | PTX+S +C | |
| W | 0.9335 | 0.9708 | |
| P value | 0.6072 | 0.8976 | |
| Passed normality test (alpha=0.05)? | Yes | Yes | |
| P value summary | ns | ns | |
|  | | | |
| Unpaired t test | |  |  |
| P value | | <0.0001 |  |
| P value summary | | **** |  |
| Significantly different (P < 0.05)? | | Yes |  |
| One- or two-tailed P value? | | Two-tailed |  |
| t, df | | t=6.412, df=10 |  |

| p-TrkB/TrkB | | | |
| --- | --- | --- | --- |
| Shapiro-Wilk test | PTX+S +V | PTX+S +C | |
| W | 0.9079 | 0.9310 | |
| P value | 0.4230 | 0.5875 | |
| Passed normality test (alpha=0.05)? | Yes | Yes | |
| P value summary | ns | ns | |
|  | | | |
| Unpaired t test | |  |  |
| P value | | 0.0037 |  |
| P value summary | | ** |  |
| Significantly different (P < 0.05)? | | Yes |  |
| One- or two-tailed P value? | | Two-tailed |  |
| t, df | | t=3.763, df=10 |  |

| Ratio of PSD95/GAPDH | | | |
| --- | --- | --- | --- |
| Shapiro-Wilk test | PTX+S +V | PTX+S +C | |
| W | 0.8434 | 0.9482 | |
| P value | 0.1390 | 0.7259 | |
| Passed normality test (alpha=0.05)? | Yes | Yes | |
| P value summary | ns | ns | |
|  | | | |
| Unpaired t test | |  |  |
| P value | | 0.0026 |  |
| P value summary | | ** |  |
| Significantly different (P < 0.05)? | | Yes |  |
| One- or two-tailed P value? | | Two-tailed |  |
| t, df | | t=3.989, df=10 |  |
