# Supplementary material for: S-ketamine mitigates paclitaxel-induced pain-related anxiety-like behavior through downregulation of mGluR5 and activation of the BDNF/TrkB signaling pathway
Source: Front Neurol. 2026 Apr 23;17:1801549. doi: 10.3389/fneur.2026.1801549 (PMC13149193; doi:10.3389/fneur.2026.1801549)
Supplement: Supplementary file 3 [file Table_3.docx]

Uncropped gels for western Blots in Figure3E

BDNF

mGluR

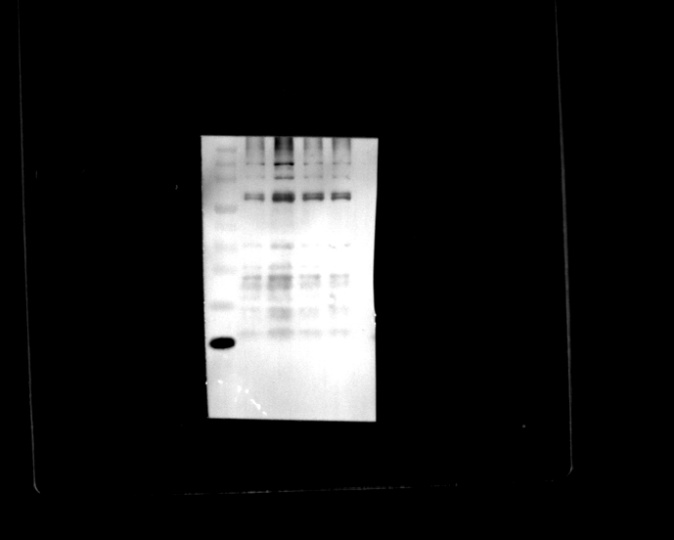

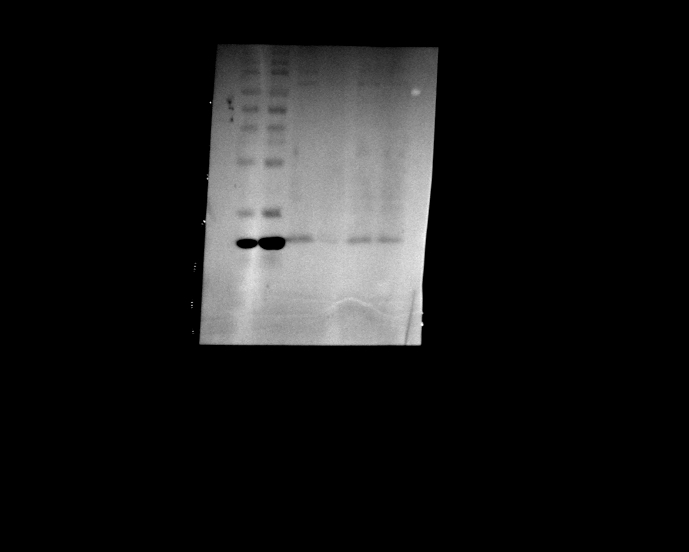


<14kDa

<132.0 kDa


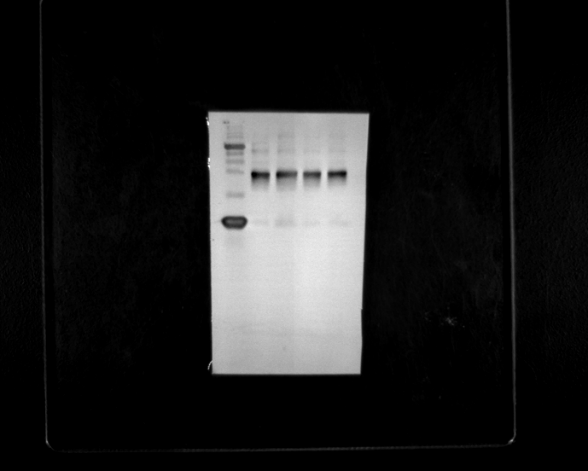

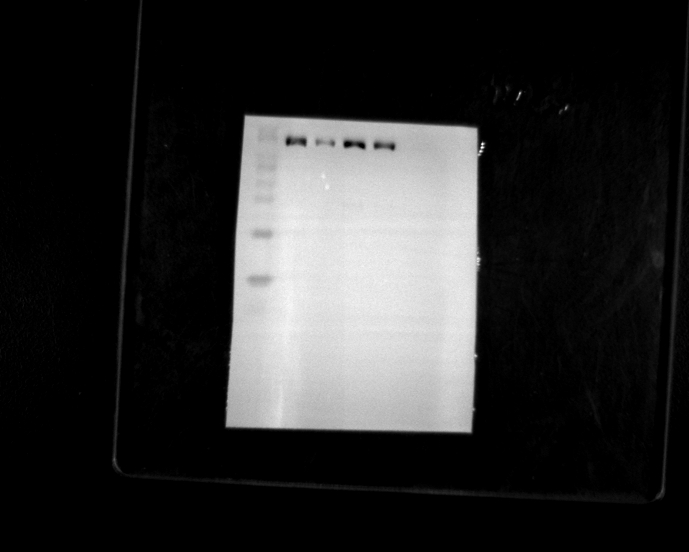

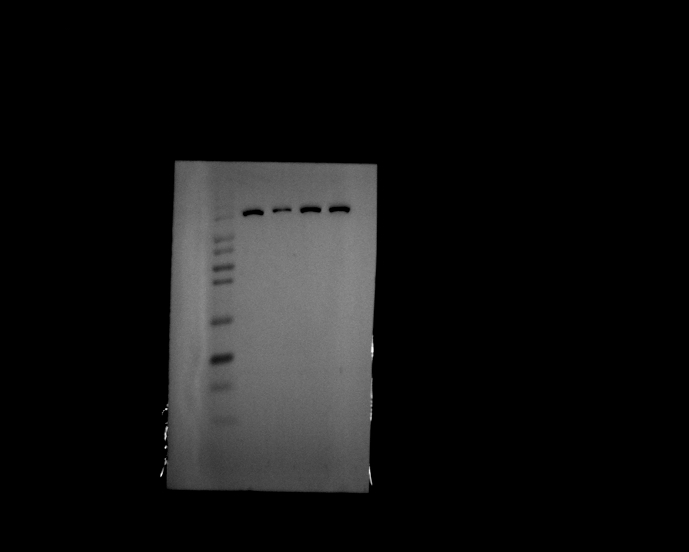

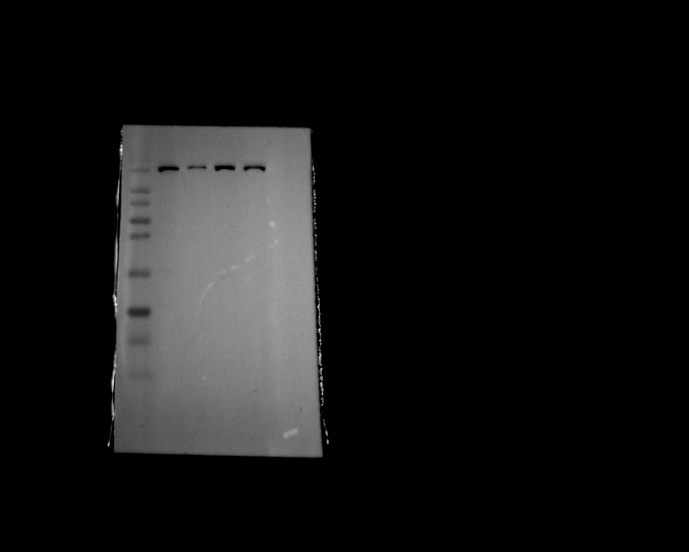


<36.0 kDa

GAPDH

<90.0 kDa

PSD95

p-TrkB

TrkB

<92.0 kDa

<92.0 kDa

Uncropped gels for western Blots in Figure5O

TrkB

BDNF

mGluR

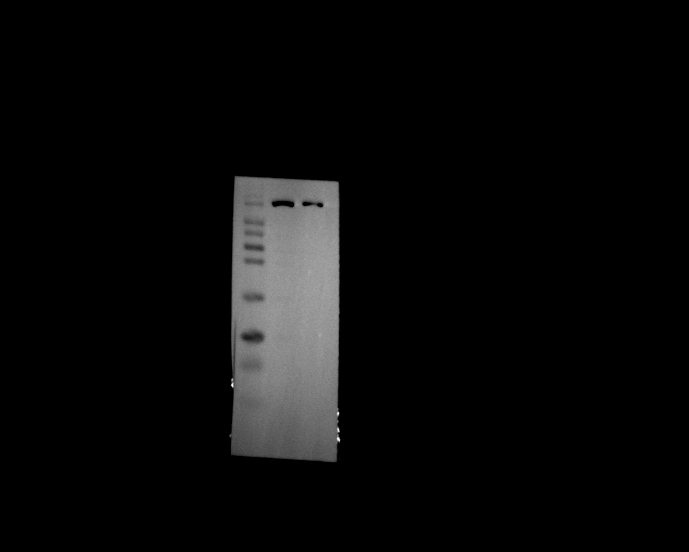

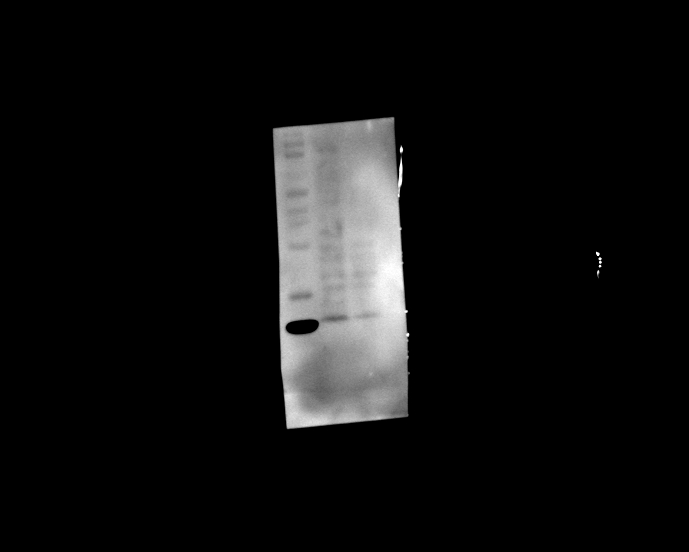

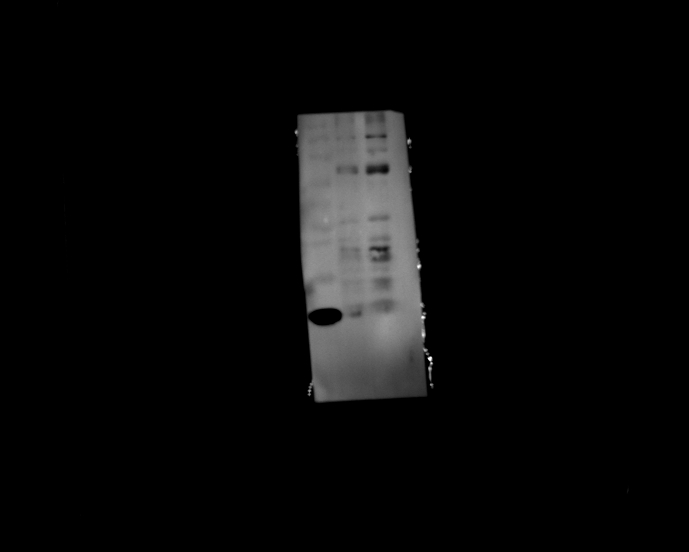


<92.0 kDa

<14kDa

<132.0 kDa

GAPDH

PSD95

p-TrkB


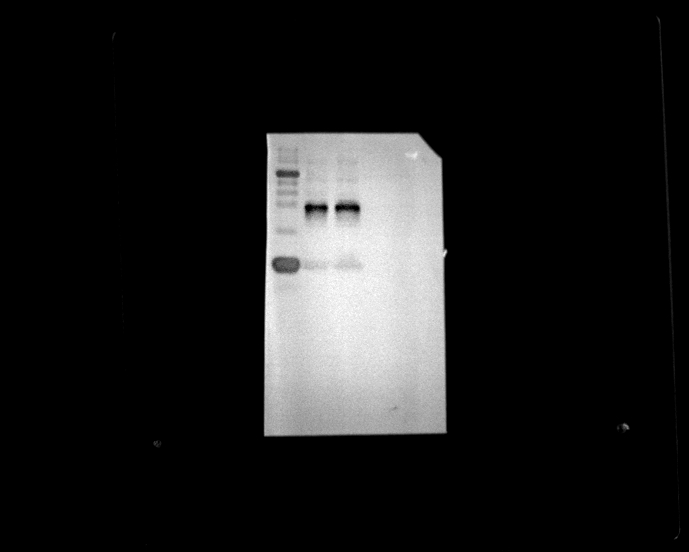

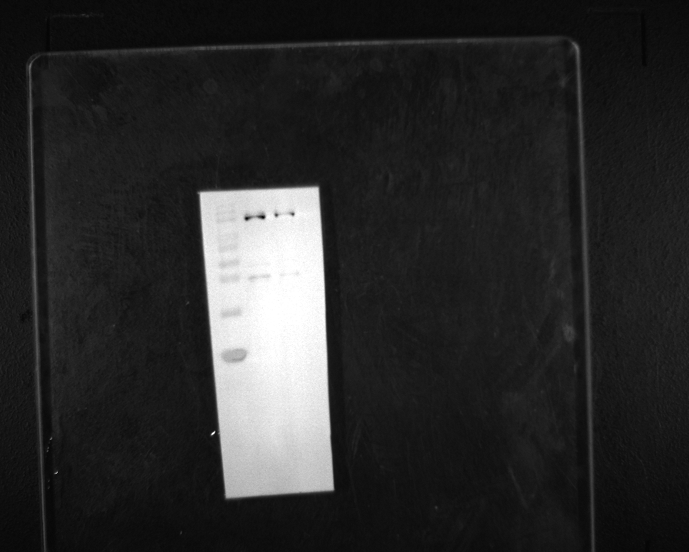

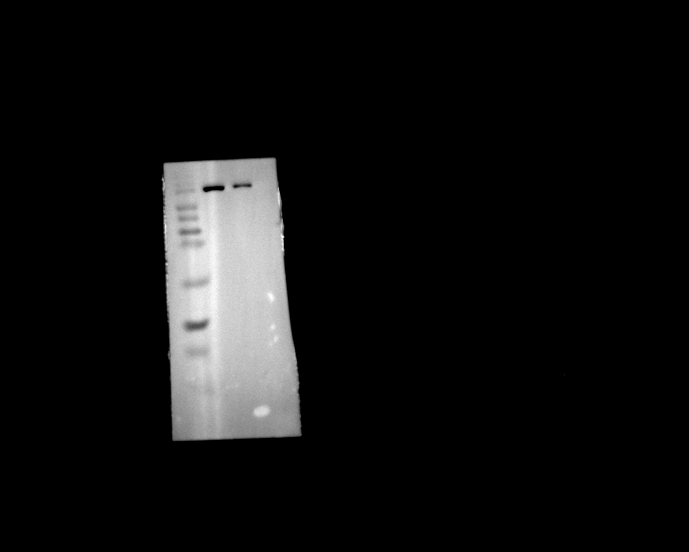


<36.0 kDa

<90.0 kDa

<92.0 kDa
